# Aryl hydrocarbon receptor suppresses STING-mediated type I IFN expression in triple-negative breast cancer

**DOI:** 10.1038/s41598-024-54732-3

**Published:** 2024-03-08

**Authors:** Jeffrey C. Martin, Tatiane da Silva Fernandes, Kanita A. Chaudhry, Masanori Oshi, Scott I. Abrams, Kazuaki Takabe, Spencer R. Rosario, Anna Bianchi-Smiraglia

**Affiliations:** 1 Department of Cell Stress Biology, Roswell Park Comprehensive Cancer Center, Buffalo, NY USA; 2 Department of Breast Surgery, Roswell Park Comprehensive Cancer Center, Buffalo, NY USA; 3 Department of Immunology, Roswell Park Comprehensive Cancer Center, Buffalo, NY USA; 4 Department of Biostatistics and Bioinformatics, Roswell Park Comprehensive Cancer Center, Buffalo, NY USA; 5 Department of Pharmacology and Therapeutics, Roswell Park Comprehensive Cancer Center, Buffalo, NY USA

**Keywords:** Aryl hydrocarbon receptor, Triple-negative breast cancer, BRCA1/2 mutations, PARP inhibitors, cGAS-STING, Type I interferons, Breast cancer, Cell signalling

## Abstract

Triple-negative breast cancer (TNBC) is one of the most aggressive types of cancer. Despite decades of intense investigation, treatment options remain limited, and rapid recurrence with distant metastases remains a significant challenge. Cancer cell-intrinsic production of cytokines such as type I interferons (IFN-I) is a known potent modulator of response to therapy in many cancers, including TNBC, and can influence therapeutic outcome. Here, we report that, in TNBC systems, the aryl hydrocarbon receptor (AhR) suppresses IFN-I expression via inhibition of STImulator of Interferon Genes (STING), a key mediator of interferon production. Intratumoral STING activity is essential in mediating the efficacy of PARP inhibitors (PARPi) which are used in the treatment of cancers harboring BRCA1 deficiency. We find that, in TNBC cells, PARPi treatment activates AhR in a BRCA1 deficiency-dependent manner, thus suggesting the presence of a negative feedback loop aimed at modulating PARPi efficacy. Importantly, our results indicate that the combined inhibition of PARP and AhR is superior in elevating IFN-I expression as compared to PARPi-alone. Thus, AhR inhibition may allow for enhanced IFN-I production upon PARPi in BRCA1-deficient breast cancers, most of which are of TNBC origin, and may represent a therapeutically viable strategy to enhance PARPi efficacy.

## Introduction

Triple-negative breast cancer (TNBC) is a highly aggressive form of breast cancer that lacks the expression of targetable molecular features, mainly ER, PR and HER2 receptors^1^. These tumors account for roughly 15–20% of all breast cancer cases and due to the relative lack of consistent genetic or molecular features, treatment options remain limited and mainly consists of surgery and highly toxic chemotherapy^2^. Furthermore, TNBC displays faster rates of recurrence with distant metastasis following initial treatment of any breast cancer subtype and is associated with the worst prognosis^2,3^. Throughout the course of the two most recent decades, there has been a concerted effort aimed at the identification of novel therapeutic options for this disease. Importantly, the use of immunotherapy (atezolizumab, an anti-PD-L1 monoclonal antibody) in combination with chemotherapy was recently approved for use as first-line therapy in metastatic TNBC tumors that overexpress PD-L1, owing to the potential for immunomodulation as a therapeutic opportunity for this indication^4^. However, only a fraction of patients responds to immune checkpoint blockade and most of these patients will relapse with distal recurrent disease, highlighting the importance to improve therapeutic responses^5^.

Tumors that harbor defects in homologous recombination repair (HRR), mainly homozygous BRCA1/2 mutations, have benefited from the introduction of inhibitors of the Poly-ADP Ribose Polymerase-1 (PARP1), an enzyme involved in DNA break repair^6^. The majority of hereditary BRCA-associated breast cancer tumors have the molecular features of TNBC^7^ and typically receive a regimen of chemotherapy centered around the use of PARP1 inhibitors (PARPi)^8^. While the use of PARPi has significantly improved the progression-free survival of patients with advanced HRR-deficient ovarian cancer^9^, the long-term response rates among breast cancer patients remain suboptimal^10^. Understanding the molecular mechanisms behind the lack of PARPi efficacy in breast cancer could lead to the identification of novel therapeutic combinations.

Several recent studies have suggested a mechanism in which, upon PARPi treatment, the cancer cells induce alterations to the tumor microenvironment (TME), particularly the tumor-infiltrating lymphocytes, and these changes appear to be essential in mediating PARPi anti-tumor response in BRCA1-mutated breast cancer tumors and the overall efficacy of PARPi^11–13^. Specifically, PARPi-induced genetic instability has been shown to activate the cytosolic pattern recognition receptor cGAS and its associated pathway STImulator of Interferon Genes (cGAS-STING^14^) within the BRCA1-mutant cancer cells, resulting in the initiation of type I IFN production, cytokine-mediated T cell infiltration, and subsequent anti-tumor immune response^11^. Interestingly, STING activity is suppressed in several cancer types, likely attributed to its role as a tumor suppressor^15^. Therefore, improving the PARPi-mediated STING activation and subsequent type I interferon (IFN-I) expression may lead to enhanced immunogenicity of PARP inhibitors in breast cancer, improving overall efficacy^11,12^.

The aryl hydrocarbon receptor (AhR) is a ligand-activated transcription factor and a member of the bHLH superfamily of transcription factors. Initially discovered as the main mediator of xenobiotics metabolism and clearance, it has subsequently become apparent that AhR can respond to a variety of endogenous cellular ligands that mediate its functions^16–18^. Active AhR harbors a potent ability to influence gene expression dynamics at both the genetic and epigenetic levels^19–22^. In the inactive form, AhR is kept sequestered in the cytoplasm in complex with chaperone proteins. Ligand binding triggers its nuclear localization where it dissociates from its retention complex and dimerizes with its transcriptional coactivator ARNT. AhR-ARNT then facilitates the recruitment of transcription complexes to several loci throughout the genome^18^. Among the most prominent AhR target genes are the Cytochrome P450, family 1, subfamily A/B, polypeptide 1 (*CYP1A1* and *CYP1B1*), which are crucial players in the metabolism of xenobiotic agents such as dioxin^23^. Importantly, AhR is known to be a potent modulator of immunity and has consequential roles in several disease settings including autoimmunity and cancer^24–31^.

In several tumor types, the production of endogenous AhR ligands accelerates during disease progression, resulting in elevated intratumoral AhR activity in advanced disease. The consequences of elevated AhR activity are context dependent as it has been shown to harbor both pro- and anti-tumorigenic functions. For example, AhR has been shown to have tumor suppressor activity in various cancer types including intestinal, prostate, lung, and liver (reviewed in^32^). In breast cancer, AhR suppresses the transcriptional activity of ER, thereby inhibiting hormone-mediated proliferation and hampering the disease progression of ER + breast tumors^33^. Furthermore, AhR ligands have been shown to augment chemotherapy efficacy, enhancing the anti-tumor effects of treatment^34–36^. On the other hand, AhR activity is known to enhance pro-tumor functions such as invasion, migration^20,37^, resistance to apoptosis^38^, and cancer stem cell development^19^ in various tumor types. AhR activity can also influence the immune microenvironment, where it promotes an immunosuppressive TME, favoring disease progression^16^. Importantly, several recent studies have revealed a role for AhR in the inhibition of cGAS-STING pathway activity^39–42^. Direct investigations aimed at elucidating the precise role of AhR in specific disease settings are needed to fully understand its role in cancer. In line with this notion, the consequences of interfering with AhR signaling with respect to STING activity and immunomodulation in TNBC has not yet been investigated to date.

Here, we provide evidence that AhR activity suppresses STING-mediated IFN-I expression in a panel of TNBC cell lines. Additionally, we identify a novel AhR-mediated negative feedback loop that modulates PARPi-induced IFN-I expression in BRCA1-deficient TNBC cells. We found that PARPi treatment activates AhR, a phenomenon further accentuated in the context of BRCA1-deficiency, resulting in the induction of key AhR target genes among which is *TIPARP*, a known antagonist of STING-induced IFN response^39^. Consistently, we found that addition of an AhR antagonist augmented the IFN-I expression upon PARPi treatment in a cell line model of BRCA1-deficient TNBC. Thus, our study identifies AhR as a promising target to elevate PARPi-induced IFN-I expression, potentially enhancing the immunogenicity and overall efficacy of PARPi in HRR-deficient TNBC.

## Results

### AHR levels associate with poor survival in TNBC tumors

**Figure 1:**
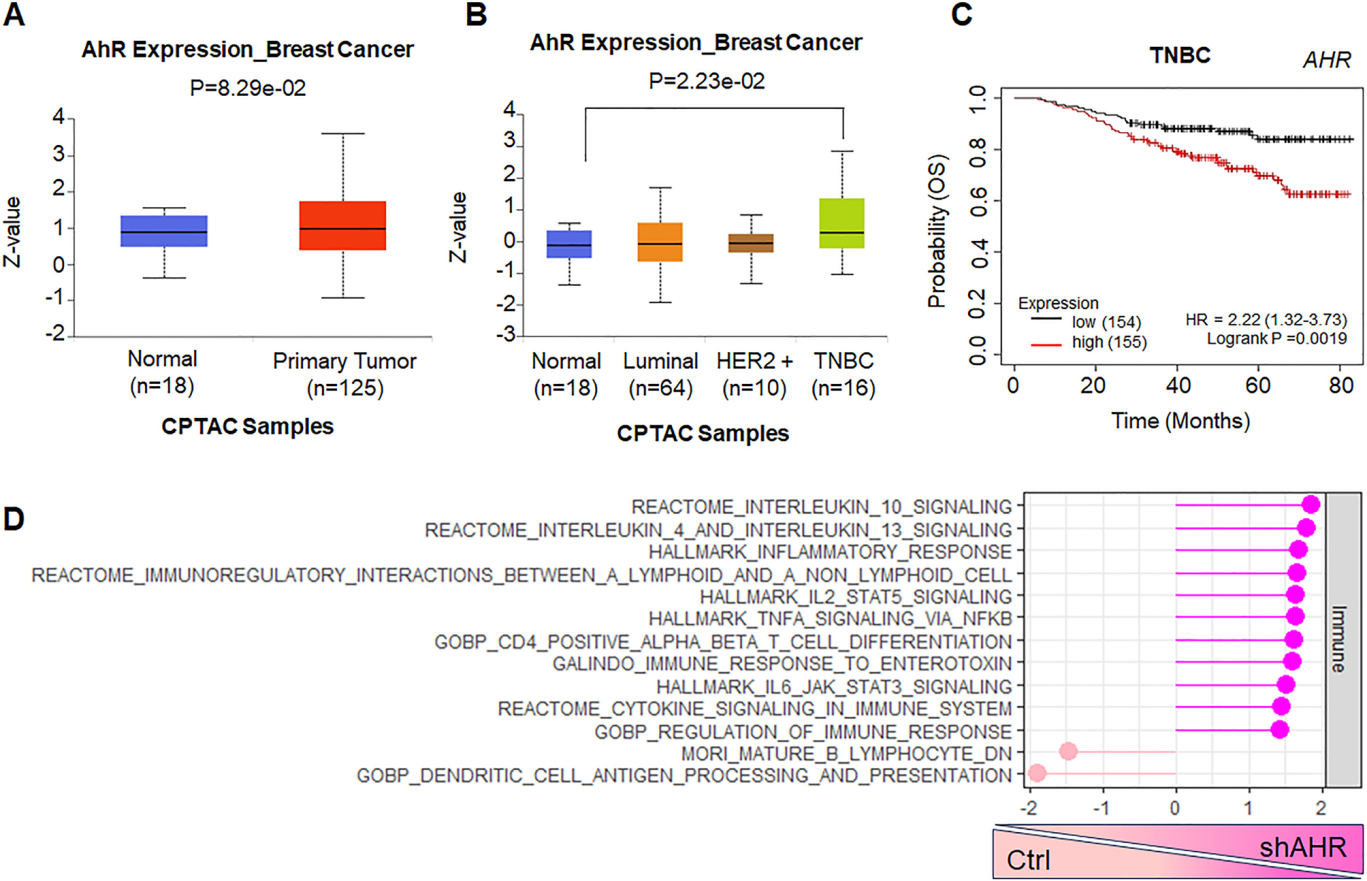
AHR is a negative prognostic indicator in triple-negative breast cancer. (**A**) Expression levels of AhR protein in Normal and Primary Breast Cancer Tumor Samples. Analysis performed at https://ualcan.path.uab.edu using CPTAC samples. (**B**) Analysis as in A but with separation of the breast cancer cases into the four major subtypes. Statistics by Welch’s T-test. (**C**) Kaplan Meier analysis for overall survival of TNBC patients, stratified on median level of AHR expression performed at https://kmplot.com. Statistics by LogRank Test. (**D**) GSEA pathway enrichment analysis of DEGs from MDA-MB-231 shAHR RNA-seq for several Immune-related pathways.

AhR has been proposed to act as both a tumor suppressor^32,43^ and tumor promoter^19,20,37,44^ in breast cancer. To better understand the role of AhR in breast cancer, we queried the University of Alabama at Birmingham Cancer (UALCAN) data analysis portal^45^, which hosts a collection of publicly available cancer omics, including protein data from the Clinical Proteomic Tumor Analysis Consortium (CPTAC)^46–48^. The analysis revealed that AhR protein levels are slightly increased in the primary breast tumor cohort as compared to normal tissue (Fig. 1A). This differential protein abundance is mainly driven by increased levels in the triple negative (TNBC) subtype (Fig. 1B) suggesting that AhR may have a specific role in this subtype, corroborating previous reports. Consistently, Kaplan–Meier estimation, via datasets available at KMplot.com^49^, revealed that AHR levels displayed an inverse association with overall survival (OS) only in TNBC patients (Fig. 1C and Supplemental Fig. S1A–C). To further delve into the role of AhR in TNBC cells, human MDA-MB-231 cells were depleted of AHR via two independent, previously validated shRNAs^41^, and RNAseq was performed. Gene Set Enrichment Analysis (GSEA) of the differentially expressed genes revealed that AHR depletion led to an increase in inflammatory response and various immune-related pathways (Fig. 1D and Supplemental Fig. S2).

**Figure 2:**
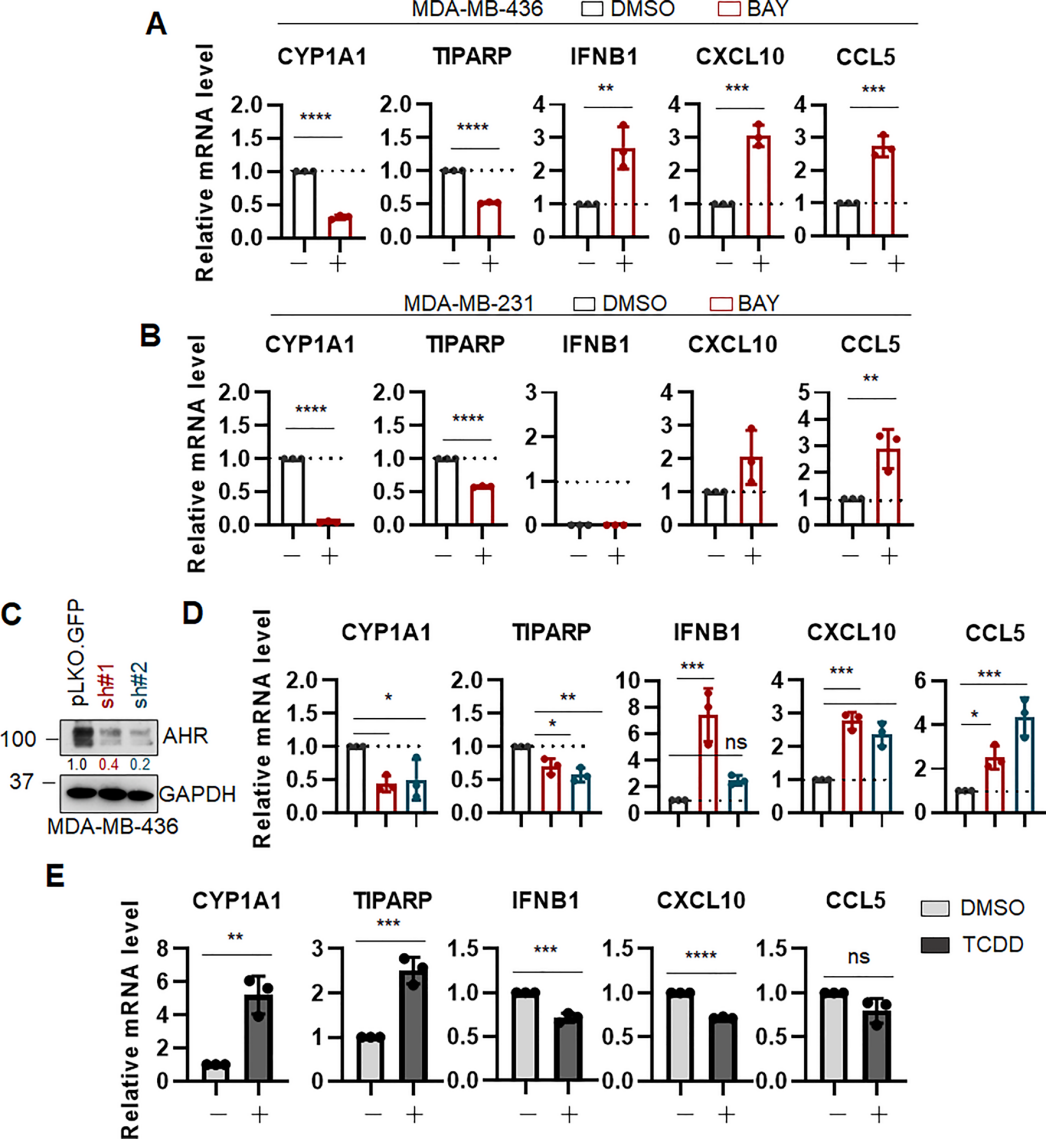
AHR suppresses Type I Interferon expression in TNBC cells. qRT-PCR analysis of indicated genes in MDA-MB-436 (**A**) and MDA-MB-231 (**B**) cells with BAY-2416964 (BAY, 20 µM, 48 h). Data (n ≥ 3 biological replicates) is average −/+ std dev. Statistics by unpaired *t* test. (**C**) Immunoblot for AhR in MDA-MB-436 cells transduced with two independent shRNA toward AHR or a non-targeting control vector (pLKO.GFP). GAPDH is used as a loading control. Quantification performed with ImageJ. Membranes were cut prior to hybridization with antibodies. (**D**) qRT-PCR analysis of indicated genes in cells as in (**C**). pLKO-GFP = black bars, shAHR #1 = red bars, shAHR #2 = blue bars. Data (n = 3 biological replicates) is average −/ + std dev. Statistics by 2-way ANOVA. (**E**) qRT-PCR analysis of indicated genes in MDA-MB-436 treated with TCDD (50 nM, 96 h) or vehicle control (DMSO). Data (n = 3 biological replicates) is average −/+ std dev. Statistics by unpaired *t* test. *p < 0.05; **p < 0.01; ***p < 0.005; ****p < 0.0005.

Altogether, these data support previous claims that AhR is detrimental for TNBC^19,20,37,44^ and suggest a potential role in suppressing immune pathways within the cancer cells.

### AhR suppresses IFN-I expression in TNBC cell lines

Recent work suggests that AhR activity antagonizes STING-mediated IFN-I expression in multiple cancer types including lung and muscle-invasive bladder cancer^39,40^. Given the increase in immune pathway-related signatures upon AHR depletion in MDA-MB-231 TNBC cell line (Fig. 1D), we asked whether these findings represented a common response in TNBC. To this end, a panel of human TNBC cell lines (MDA-MB-231, MDA-MB-436, and HCC1937) was treated with the AhR antagonists BAY-2416964 (herein referred to as BAY), which is currently being tested in clinical trials for treatment of advanced solid tumors (NCT04069026 and NCT04999202). Due to the presence of intrinsic AhR activity within these cells, potentially due to the endogenous production of AHR ligands^41,50^, the treatment resulted in reduction of AhR established targets *CYP1A1*^23^ and *TIPARP*^51^. Importantly, AHR antagonism also resulted in a significant induction of the expression of key type I IFNs, *IFNB1*, *CXCL10*, and *CCL5*, in all TNBC cell lines tested (Fig. 2A, B and Supplemental Figure S3A). Importantly, this was not observed in either the immortalized breast epithelial non-tumor MCF10A cells (Supplemental Figure S3B) or the immortalized human fibroblast cell line WI-38 (Supplemental Figure S3C), suggesting that this phenomenon could be restricted to tumor cells.

Consistent results were obtained with AhR genetic, rather than pharmacologic, suppression. MDA-MB-436 cells were depleted of *AHR *via shRNA as above (Fig. 2C) and a reduction in AhR target genes *CYP1A1* and *TIPARP* was observed (Fig. 2D) concomitantly with a significant increase in the expression of *IFNB1, CXCL10,* and *CCL5* (Fig. 2D). On the converse side, activation of AhR with its classical agonist TCDD^17^ resulted in an induction of the expression of target genes *CYP1A1* and *TIPARP* (Fig. 2E and Supplemental Figure S4A,B), as well as in a mild but very consistent reduction in the expression of *IFNB1*, *CXCL10*, and *CCL5* in MDA-MB-436 cells (Fig. 2E).

Thus, AhR has the capability of modulating the expression of IFN-I -related genes in TNBC cells.

### AhR suppresses STING-dependent IFN-I expression

**Figure 3:**
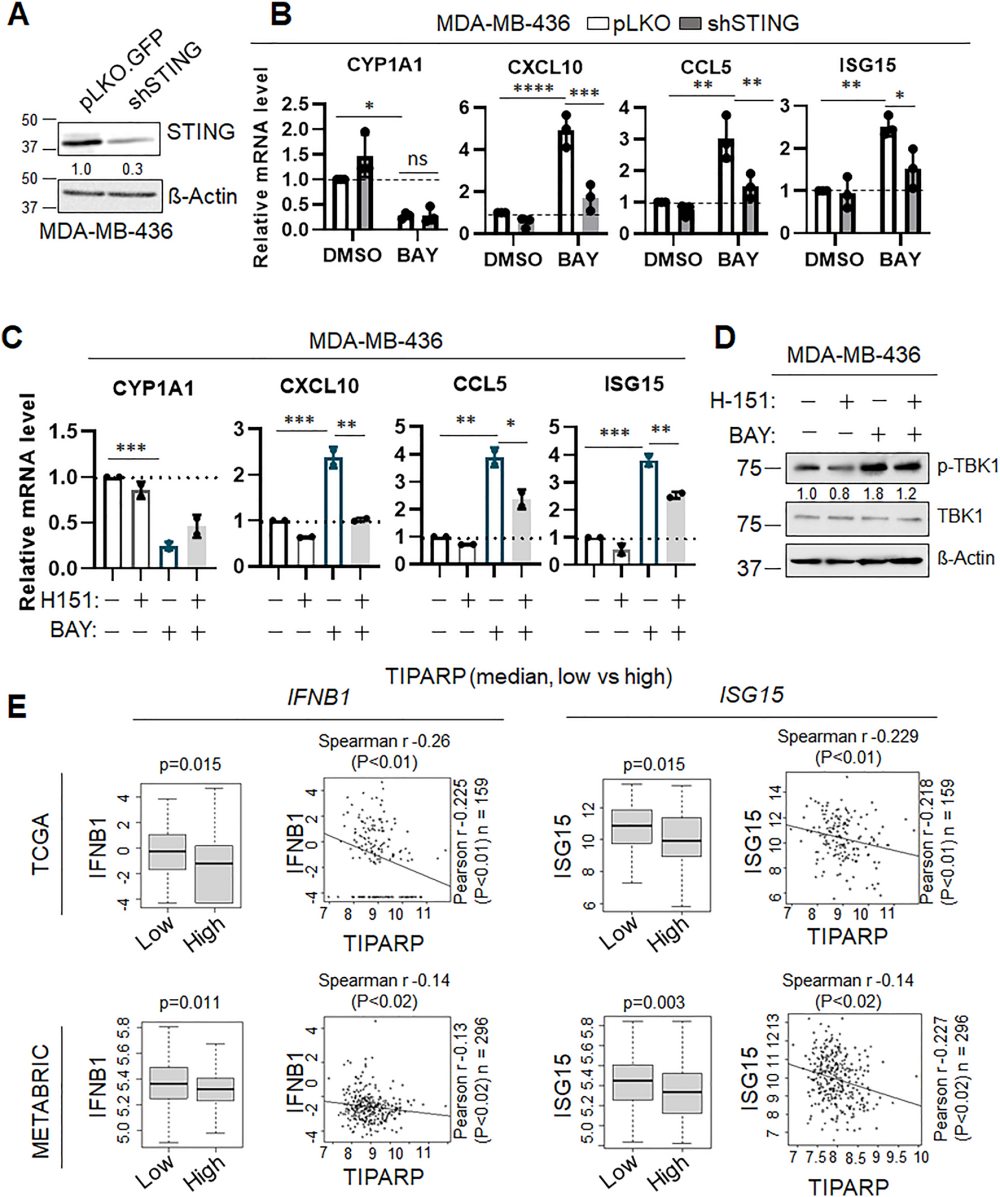
AHR antagonist-mediated IFN-I expression is STING-dependent. (**A**) Immunoblot for STING in MDA-MB-436 cells transduced with shRNA toward STING or a non-targeting control vector (pLKO.GFP). ß-Actin is used as a loading control. Quantification performed with ImageJ. Membranes were cut prior to hybridization with antibodies. (**B**) qRT-PCR analysis of indicated genes in MDA-MB-436 cells (pLKO = white bars, shSTING = grey bars – 20 µM BAY, 72 h). Data (n = 3 biological replicates) is average −/ + std dev. Statistics by 2-way ANOVA. **C.** qRT-PCR analysis of indicated genes in MDA-MB-436 cells (10 µM H-151, 20 µM BAY2416964, or the combination, 72 h). Data (n = 2 biological replicates) is average −/ + std dev. Statistics by 2-way ANOVA *p < 0.05; **p < 0.01, ***p < 0.005. (**D**) Representative immunoblot for pTBK1 (S172), total TBK1, and ß-Actin in MDA-MB-436 cells treated as in panel B. Quantification performed with ImageJ (figure shows representative image of 3 biological replicates). Membranes were cut prior to hybridization with antibodies. (**E**) Relative expression levels of *IFNB1* and *ISG15* in TCGA (top) and METABRIC (bottom) databases in patients split on *TIPARP* median levels*.* Statistics by Wilcoxon signed-rank test.

TNBC tumors are known to exhibit high genomic instability, and therefore high likelihood of enhanced basal cGAS-STING activity^15^. AhR has been proposed to have antagonistic functions toward STING in other cancer types^39,40^. Thus, to assess whether the changes in IFN-I genes we observed upon AhR manipulation in TNBC are dependent on STING activity, we took a two-fold approach as above. STING was genetically depleted in the MDA-MB-436 cells using shRNAs (Fig. 3A); empty vector control (pLKO) cells and STING-knockdown cells were treated with BAY for 72 h and the expression of IFN-I genes was assessed. As before, a significant increase in *IFNB1*, *CXCL10* and *CCL5* expression, as well as the interferon-stimulated gene ISG15, was observed upon BAY treatment in control cells (Fig. 3B and Supplemental Figure S5A). However, this increase was blunted by STING depletion (Fig. 3B and Supplemental Fig. S5A), suggesting that BAY-mediated IFN-I expression is STING-dependent. These results were then confirmed using the STING-specific small molecule inhibitor H-151^52^. Similarly to the genetic depletion, inhibition of STING with H-151 suppressed the interferon gene induction promoted by BAY (Fig. 3C and Supplemental Figure S5B). Furthermore, immunoblot analysis of cells treated as above revealed that AhR inhibition with BAY induced TBK1 phosphorylation at S172, an event known to be STING-dependent^53^ and necessary to mediate IFN gene production in response to STING activation^15^. Concomitant treatment with H-151 attenuated this effect (Fig. 3D and Supplementary Figure S5C).

To gain more insight into the relationship between AhR and STING activity in TNBC tumors, we analyzed the TCGA and METABRIC datasets by parsing expression of known AHR target genes and known STING pathway-associated genes. The analysis revealed inverse expression levels between *TIPARP* (a key target of AhR) and *IFNB1* or *ISG15* (known to be influenced by STING activity^14^ (Fig. 3E), suggesting that tumors with high AhR activity (represented by high *TIPARP*) may have reduced interferon-based response.

Taken together, these results support previous findings that AhR antagonism increases IFN-I expression, and expand on this concept, by proposing a STING-dependent mechanism.

### AhR suppresses PARPi-induced IFN-I expression

**Figure 4:**
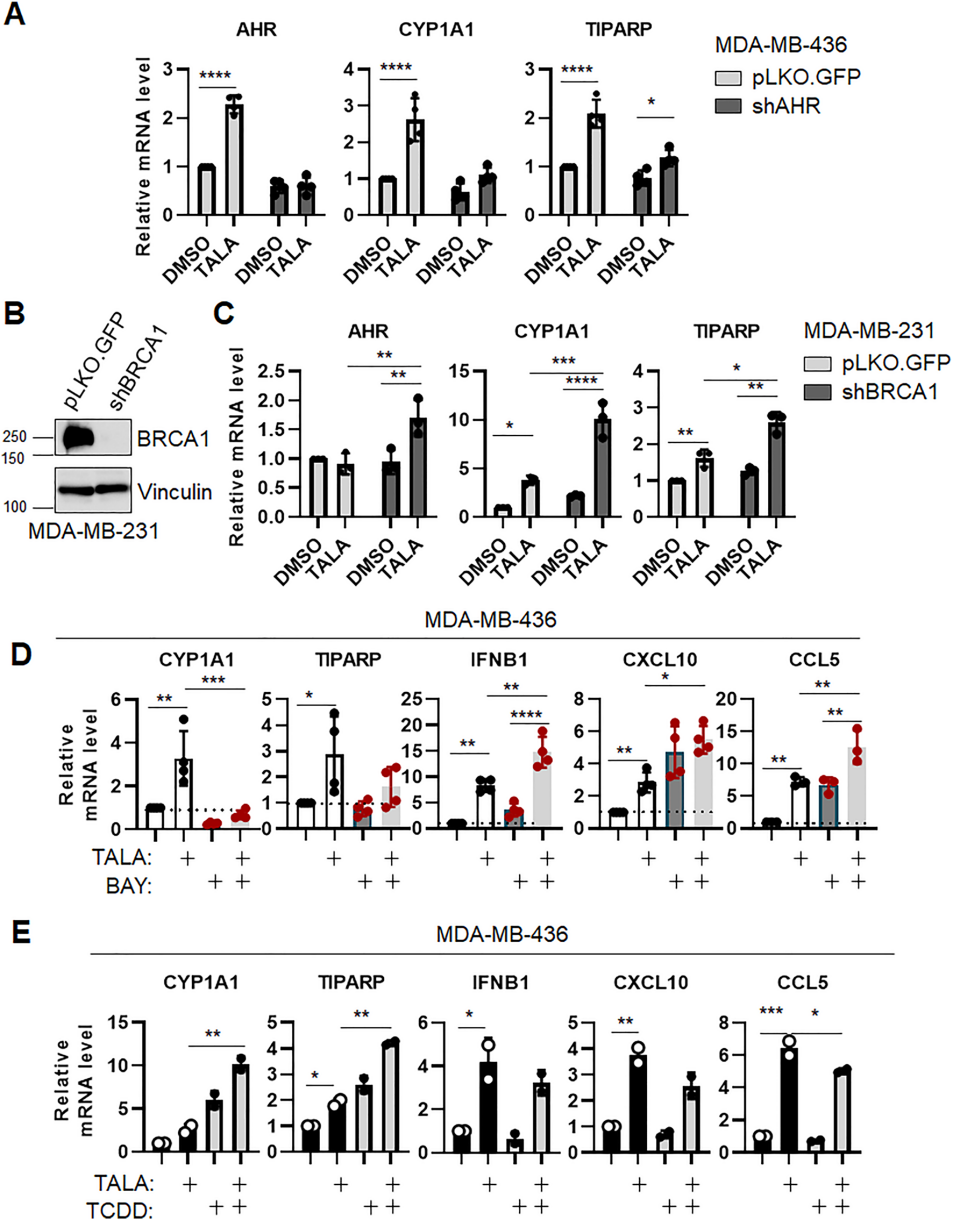
AHR suppresses PARPi-induced IFN-I expression in BRCA1-deficient TNBC. (**A**) qRT-PCR analysis of indicated genes in MDA-MB-436 cells (pLKO = light grey bars, shAHR #2 = dark grey bars—1 µMTalazoparib, 96 h). Data (n = 4 biological replicates) is average −/ + std dev. Statistics by 2-way ANOVA. (**B**) Immunoblot for BRCA1 in MDA-MB-231 cells transduced with shRNA toward BRCA1 or a non-targeting control vector (pLKO.GFP). Vinculin is used as a loading control. Membranes were cut prior to hybridization with antibodies. (**C**) qRT-PCR analysis of indicated genes in MDA-MB-231 cells (pLKO = light grey bars, shBRCA1 = dark grey bars—1 µMTalazoparib, 96 h). Data (n = 3 biological replicates) is average −/ + std dev. Statistics by 2-way ANOVA. (**D**) qRT-PCR analysis of indicated genes in MDA-MB-436 cells (1 µM Talazoparib, 20 µM BAY2416469, or the combination, 96 h). Data (n ≥ 3 biological replicates) is average −/ + std dev. Statistics by 2-way ANOVA. (**E**) qRT-PCR analysis of indicated genes in MDA-MB-436 cells (1 µM Talazoparib, 50 nM TCDD, or the combination, 96 h). Data (n = 2 biological replicates) is average −/ + std dev. Statistics by 2-way ANOVA. *p < 0.05; **p < 0.01, ***p < 0.005, ****p < 0.0005.

STING-mediated production of IFN-I in a cancer cell-intrinsic fashion has been recently shown to be essential for the anti-tumor effects of PARPi treatment in BRCA1-deficient TNBC^11^. To assess whether any relationship exists between PARPi and AhR, the BRCA1-mutant MDA-MB-436 cells^54^ were transduced with control vector or shAHR and treated with the PARP inhibitor Talazoparib (an FDA approved PARP inhibitor currently being used in the clinic for BRCA1/2-mutant TNBC and ovarian cancer^55^; herein referred to as TALA). Surprisingly, we found that TALA treatment in these cells resulted in AhR induction and an AhR-dependent increase of its target genes *CYP1A1* and *TIPARP*, thus suggesting that PARPi induces AhR activation (Fig. 4A). To assess whether this phenomenon was dependent on BRCA1 activity, we expanded to a second cell line model by performing BRCA1-knockdown via shRNA in MDA-MB-231 cells, a well-established BRCA1^WT^ TNBC^54^ cell line (Fig. 4B) and treating cells with TALA. As expected, shBRCA1 MDA-MB-231 cells displayed increased sensitivity to PARP inhibition (Supplemental Figure S6)^6^. Importantly, TALA treatment caused a robust induction in expression of *AHR, CYP1A1,* and *TIPARP* in the BRCA1 knockdown cells (Fig. 4C), while these effects were markedly reduced in control cells.

Thus, our data suggest that in conditions of BRCA deficiency, PARP inhibition results in AhR activation. This, combined with our previous data, leads to a model in which AhR activation by PARPi may create a self-regulatory loop that suppresses PARPi induction of IFN-I genes, thereby alleviating the effects of PARP inhibition.

To test this, MDA-MB-436 cells were treated with either BAY (AhR antagonist), TALA (PARP inhibitor), or a combination of the two and the expression of AhR targets and IFN-I genes was assessed. As before, TALA by itself induced *CYP1A1* and *TIPARP*, which could be suppressed by concomitant BAY treatment (Fig. 4D, first two panels) and both BAY and TALA induced increases in the levels of IFN-I genes *IFNB1, CXCL10,* and *CCL5* (Fig. 4D, last three panels). However, the combination of TALA + BAY resulted in a greater induction of these IFN-I genes as compared to either drug alone, thus suggesting that AhR antagonism may potentiate the effects and durability of PARPi. Conversely, AhR activation with its prototypical ligand TCDD, resulted in the opposite effect, blunting the induction of IFN-I genes by TALA (Fig. 4E).

Altogether, our work reveals a novel function for AhR in the suppression of tumor-intrinsic, STING-mediated IFN-I production and helps to shed light on the lack of efficacy of PARPi in TNBC.

## Discussion

**Figure 5:**
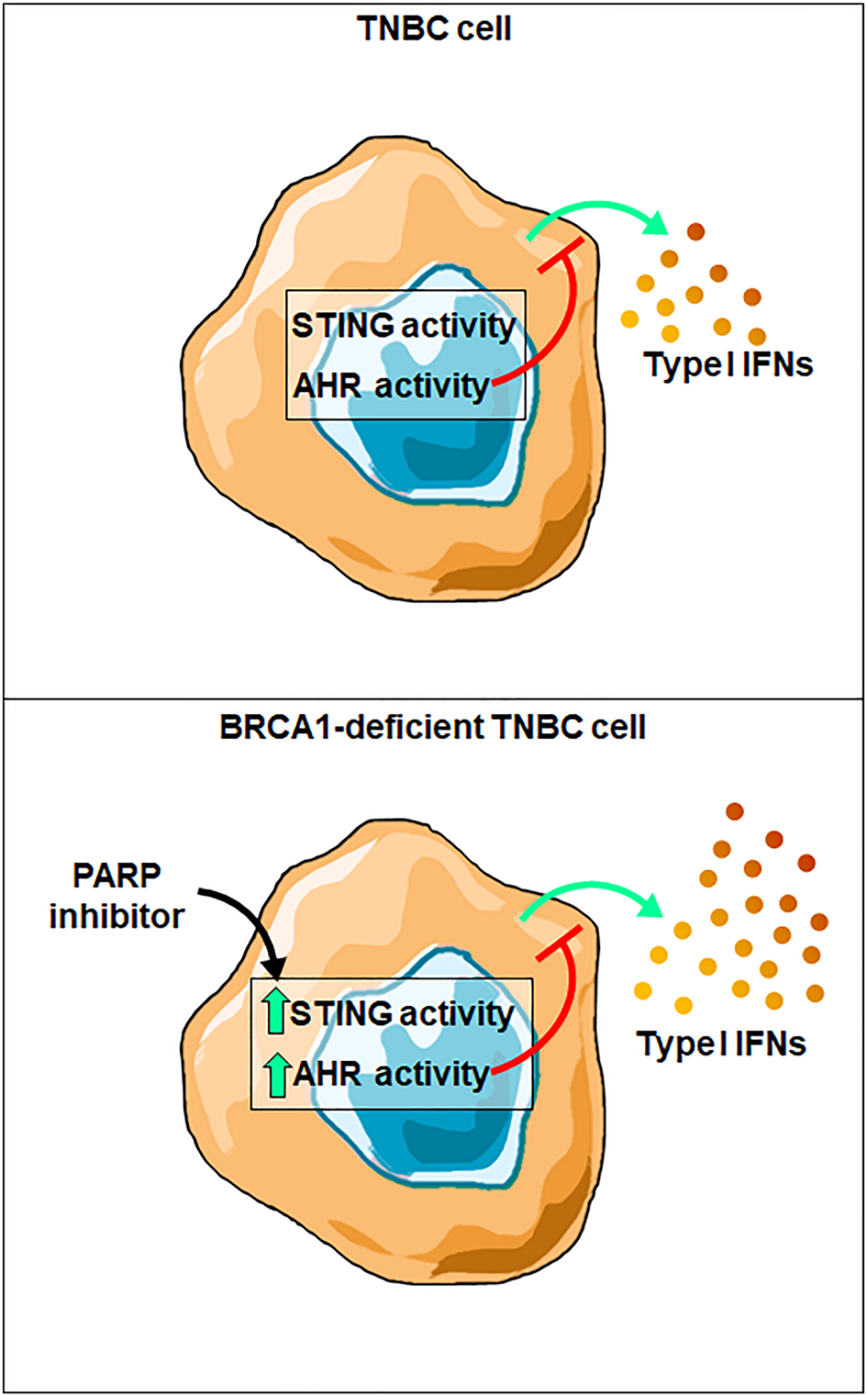
Model of AhR suppression of STING-mediated Type I IFN expression upon PARPi. (TOP) In TNBC cells under basal conditions, AHR activity suppresses STING-mediated IFN-I production. (BOTTOM) In BRCA1-deficient TNBC cells, PARPi stimulates STING activity but at the same times it induces AhR activation, which in turns acts as a feedback mechanism to reduced STING activity and its induction of Type I interferon genes.

The cGAS-STING pathway has received much attention as a promising immunomodulatory therapeutic target because it represents a mechanistic link between cellular stress, a hallmark of cancerous cells and chemotherapeutic mechanisms of action, and the activation of the innate immune system^14,15,53,56,57^. Recent findings highlight a key role for this pathway in mediating anti-tumor immunity and immune checkpoint inhibitor efficacy^58,59^. Importantly, STING activity has also been shown to be essential for the anti-tumor effects of various chemotherapeutics^60^, including PARPi in BRCA1-deficient TNBC^12,13^. Therefore, it has been hypothesized that overall tumor immunogenicity and therapeutic efficacy, including that of PARPi, can be improved by enhancing cancer cell-intrinsic STING activation and subsequent IFN-I production. Here, we provide evidence that suggests that AhR activity suppresses STING-mediated IFN-I in TNBC, especially in the context of PARPi treatment of BRCA1-deficient cells, and that its inhibition represents a therapeutically viable method to enhance cancer cell-intrinsic STING activity, both alone and in combination with PARPi (Fig. 5).

TNBC cells are characteristically more proliferative and harbor higher levels of genomic instability than other subtypes of breast cancer ^61^. As a result, these tumors tend to have higher mutational burdens, elevated expression of neoantigens, and greater immunogenicity than other subtypes^62–64^. Furthermore, high genomic instability is known to activate cGAS-STING and lead to IFN-I production and immune cell infiltration^65^, highlighting another intrinsically immunostimulatory characteristic of TNBC tumors. Therefore, in order to remain viable, it is likely that TNBC tumors must adopt mechanisms that suppress the immune system and overcome genomic instability-associated immunity. Interestingly, AhR is known to promote immunosuppression in several tumor types through various mechanisms, including the inhibition of STING activity^28,30,31,40,66^. This may partly account for why TNBC tumors have higher levels of AhR and why high AHR expression is a negative prognostic indicator, only in TNBC (Fig. 1). It is likely that without sufficient AhR activity, anti-tumor immunity would act as a barrier to tumor formation. In line with this, RNAseq analysis upon AHR depletion in the TNBC cell line MDA-MB-231, revealed significant enrichment of immune-related signatures suggesting an AhR-mediated suppression of immunity in this context. Furthermore, gene specific qPCR showed that AhR inhibition alone was sufficient to upregulate key IFN-I genes including *IFNB1*, *CXCL10*, and *CCL5* while AhR activation suppressed them, in multiple cell line systems of TNBC, but not non-tumorigenic cell lines (Fig. 2 and Supplementary Figure S3).

The transcriptional activation of IFN-I genes involves a complex cellular regulatory network that is not limited to the cGAS-STING pathway^67^. However, recent findings have indicated a role for AhR in the suppression of STING^39–42^. For example, the AhR-target PARP7 (product of the gene *TIPARP*), can directly suppress STING activity by facilitating the degradation of TBK1, a key downstream effector kinase of STING, thereby suppressing IFN-I expression and subsequent anti-tumor immunity^39^. AhR has also been shown to regulate the synthesis of the polyamines spermine and spermidine^41^ which have recently been implicated in the attenuation of cGAS activity via alterations to the DNA ligands in the cytoplasm^42^. Additionally, a recent study showed that AhR itself may suppress IFN-I expression in muscle invasive bladder cancer by directly interacting with and mediating the degradation of STING^40^. Therefore, the upregulation of IFN-I genes upon AHR depletion or inhibition may be due to an alleviation of STING suppression. Consistent with this hypothesis, genetic and pharmacologic depletion of STING revealed that IFN-I upregulation upon AhR inhibition is STING-dependent (Fig. 3A–D). Importantly, analysis of gene expression data from patient tumors revealed a negative correlation between the key AhR target gene *TIPARP* and STING-associated IFN genes, *IFNB1* and *ISG15* indicating that AhR inhibition of STING is likely occurring in patient tumors. Further studies must be performed to identify the mechanistic basis of this regulation.

IFN-I cytokines harbor potent immunomodulatory properties and function to influence the recruitment and activity of immune cells^67^ and are known to have both pro- and anti-tumor functions^68^. Importantly, a foundational study published in 2015 analyzed hundreds of TNBC patient tumors and found that tumors with a high “immunostimulatory” gene expression signature were associated with the best prognosis and longest overall survival of any TNBC subtype^69^. One of the key characteristics of the “immunostimulatory” subtype was the enrichment for genes sets including: “JAK-STAT-associated cytokines’, “NF-kB activation by viruses”, and “Interferon Signaling”, which are all related to the cGAS-STING pathway and IFN-I gene signatures^14,15^. Furthermore, *CCL5* expression levels in TNBC have been associated with a better prognosis and increased recruitment of CD8 T cells, CD4 activated T cells, NK cells and anti-tumor macrophages^70^. These data heavily imply that IFN-I cytokines have potent anti-tumor activity in TNBC and justify the pursuit of therapeutic strategies to increase their intratumoral abundance. AhR antagonism as a method to achieve this end has not been directly assessed, and future studies are warranted.

cGAS-STING activity is vital for the anti-tumor effects of various chemotherapeutics including PARPi^11,12,60^. Specifically, PARPi-induced cGAS-STING activation within BRCA1-deficient cancer cells results in the intratumoral production of IFN-I, subsequent immune cell infiltration, and T cell-dependent anti-tumor immunity. STING-depleted cancer cells fail to elicit this response, which significantly diminishes the therapeutic effect of PARPi^11^. This implies that STING activity within the cancer cells themselves may potentially be important for the therapeutic effects of PARPi and that PARPi efficacy may be enhanced by increasing intracellular STING activity. Interestingly, we found that treatment with Talazoparib (an FDA approved PARP inhibitor currently used in the clinic^55^) activated AhR in TNBC cells, a phenomenon that was accentuated in the BRCA1-deficient setting (Fig. 4A–C). Being that AhR is a ligand-activated transcription factor with affinity to aromatic hydrocarbons^18^, it is possible that the chemical structure of Talazoparib itself could activate AhR. However, the fact that BRCA1 knockdown accentuated the activity of AhR implies some intracellular response to PARPi treatment that is augmented by BRCA loss, potentially apoptosis, is required for AhR activation. Regardless of the mechanistic basis, the fact that AhR activity is stimulated by PARP inhibition hints at the existence of a self-regulatory mechanism in which the production of IFN-I upon PARPi treatment is diminished by AhR; a mechanism that can be overcome with the addition of an AhR antagonist. Indeed, we found that the addition of the AhR antagonist BAY-2416964 enhanced the PARPi-mediated IFN-I production in a BRCA1-mutant TNBC cell line, suggesting that AhR antagonism may be a means to enhance the immunogenicity and overall efficacy of PARPi in the clinic.

Additionally, these findings have significant implications with regards to immune checkpoint inhibitor therapy, as STING-mediated IFN-I production is known to enhance ICI efficacy. Treatment with STING agonists has been tested as a way to enhance ICI therapy^14,56,57,71^; however, systemic delivery of STING agonists has failed due to poor stability of the specific agents in circulation or the emergence of severe side effects which limits the clinical viability of this approach^56,72^. Our results suggest that AhR antagonism may indirectly result in the activation of STING specifically within the TNBC cells. The use of AhR antagonists to enhance ICI therapy has not been tested to date, though AhR antagonists are currently being investigated for their immunomodulatory potential in several cancer types (NCT04069026 and NCT04999202).

Conversely, treatment with the AhR agonist TCDD suppressed the expression of STING-associated IFN-I genes (Fig. 2E). While the TCDD-induced IFN-I suppression was mild (~ 15–20% reduction) the data were very consistent. This is particularly relevant because several studies have reported that microbial metabolism contributes to the accumulation of AhR ligands such as indoles from tryptophan metabolism within the tumor microenvironment of multiple cancer types (i.e. PDAC^31^ and melanoma^25^), which mediate AhR-dependent immunosuppressive processes within the immune cells. Our data suggest that these ligands may also negatively influence intratumoral STING activity in an AhR-dependent manner. Given that STING activation is essential for proper PARPi-mediated anti-tumor efficacy, it would be interesting to investigate if microbiome-dependent AhR activity contributes to the disparities among patient responses to PARPi treatment. For example, dietary habits that alter microbiome composition, such as diets high in tryptophan ^25^, may result in an AhR-mediated STING-suppressed pro-tumorigenic microenvironment that hinders the immunogenicity of PARPi treatment affecting overall efficacy. This raises the possibility of investigating the potential to augment PARPi + ICI efficacy with dietary alterations or with the use of probiotic adjuvants to influence the composition of the microbiome towards a low AhR ligand-producing metabolic state.

Although limited by the fact that our studies have been conducted only in cell lines, our data strongly suggest that AhR activity suppresses STING-mediated IFN-I expression in TNBC, especially in the context of PARPi treatment of BRCA1-mutant cells (Fig. 5). Further implications, that will need to be address in vivo with the use of xenografts and mouse models, are that AhR antagonism may be a viable therapeutic strategy to enhance the overall immunogenicity of TNBC tumors and improve PARPi efficacy.

## Materials and methods

### Cell lines

MDA-MB-436 and HCC1937 cells were a kind gift from Dr. Eric Knudsen (Roswell Park Comprehensive Cancer Center, Buffalo, NY, USA). HEK-293T cells were a kind gift from Dr. Irwin Gelman (Roswell Park Comprehensive Cancer Center, Buffalo, NY, USA). MDA-MB-436 and HCC1937 cells were cultured in RPMI media (Invitrogen, Carlsbad, CA, USA). WI-38, MDA-MB-231 and HEK293T were cultured in DMEM media (Invitrogen). Both media were supplemented with 10% fetal bovine serum (Invitrogen) and 1% antibiotic–antimycotic (Invitrogen). MCF10A cells were incubated in a 50/50 DMEM/F-12 formulation (Invitrogen #11330-032) with the following supplements: Horse serum (Invitrogen #16050-122; 5% final), EGF (20 ng/mL final), Hydrocortisone (0.5 mg/mL final), Cholera Toxin (100 ng/mL final), antibiotic–antimycotic (1% final). All cell lines were authenticated via short tandem repeat (STR) sequencing at the Roswell Park Genomics Shared Resource and routinely tested for mycoplasma contamination.

### Antibodies and other reagents

#### Primary antibody information

Rabbit monoclonal antibodies to AHR ((D5S6H) Cat. No. 83200S), GAPDH ((D4C6R) Cat. No. 97166T), pTBK1/NAK (S172) ((D52C2) XP (R) Cat. No. 5483T), and TBK1/NAK (Cat. No. 3013S) were purchased from Cell Signaling (Cell Signaling Technology, Danvers, MA, USA). Mouse monoclonal antibodies to BRCA1 ((D-9) Cat. No. sc-6954), and Vinculin ((7F9) Cat. No. sc-73614) were purchased from Santa Cruz (Santa Cruz, CA, USA). Mouse monoclonal antibody to ß-Actin (Cat No. 66009-1-1g) was purchased from Proteintech (Rosemont, IL, USA). αMouse-HRP secondary antibody (Goat Anti-Mouse IgG (H + L)-HRP Conjugate cat. No. 172-1011) and αRabbit-HRP rabbit secondary antibody (Goat Anti-Rabbit IgG (H + L)-HRP Conjugate cat. No. 170-6515) were purchased from Bio-Rad (Bio-Rad, Hercules, CA, USA). AhR antagonist BAY-2416964 (Cat. No.: HY-135829), PARP inhibitor Talazoparib (No. S7048), and STING inhibitor H-151 (cat. No. S6652) were purchased from Selleckchem (Houston, Texas, USA).

### Cell viability assay

Cells were seeded (3000–5000 cells per well in a 6-well plate) and allowed to incubate overnight at 37 °C and treated with increasing concentrations of Talazoparib for 72 h. At the end of treatment, 10µL of Cell counting kit-8 (WST-8/CCK8; abcam cat. No. ab228554) reagent was added to each well and cells were incubated for 1–3 h. Total absorbance was measured at 450 nm on a SpectraMax M2/M2E microplate reader (Molecular Devices). Cell viability was calculated relative to the average of DMSO control-treated cells.

### Lentiviral Infections (shRNA)

shRNA plasmids were transfected into HEK293T cells using LipoD293 (SignaGen, Frederick, MD, USA) along with the pCMV-VSV-G vector (Addgene, Cambridge, MA, USA) and the pCMV-psPAX2 vector (a kind gift from Dr. Irwin Gelman, Roswell Park Comprehensive Cancer Center, Buffalo, NY, USA). pLKO-GFP vector was purchased from Sigma. All shRNA expressing plasmids were purchased from Sigma Aldrich: shAHR #1 TRCN0000245285; target sequence: 5’ GCAACAAGATGAGTCTATT 3’, shAHR #2 TRCN000021258; sequence: 5’ CGGCATAGAGACCGACTTA 3’, shSTING (TMEM173) TRCN0000134594; target sequence: 5’ GTTTACAGCAACAGCATCTAT 3’, shBRCA1 TRCN0000009823; target sequence: 5’ CTCTTAAAATATAAGACCTCTGGCATGAAT 3’. All lentiviral infections were performed as previously described^41^. Briefly, lentiviral supernatant was harvested at 48 h post transfection and transduced to cells in the presence of 8 $\upmu$g/mL hexadimethrine bromide (Sigma, St. Louis, MO, USA). Cells were incubated with virus for 12 h, followed by a 24-h incubation with non-virus growth media. Cells were then subjected to puromycin selection for 48–72 h. PURO-resistant surviving cells were aggregated and used for further experimentation.

### Real time quantitative PCR (RT-qPCR)

Total cellular RNA was collected using the PureLink™ RNA Mini (ThermoFisher Scientific, Waltham, MA, USA cat. No. 12183025) with on-column DNase treatment, as per manufacturer’s recommendations. 1 μg of total RNA was reverse transcribed using the High-Capacity cDNA Reverse Transcription Kit (Applied Biosystems Waltham, MA, USA cat. No. 4368814). Quantitative real time PCR was performed on a CFX Opus 96 Real-Time PCR System (Biorad, Hercules, CA, USA Cat. No. #12011319) using iTaq Universal SYBR® Green Supermix (Biorad, Hercules, CA, USA. Cat. No. 1725121) with the following human site-specific primers listed below. Data were analyzed using the Bio-Rad CFX Maestro software.

RPS20 FWD: AAGGATACCGGAAAAACACCC; RPS20 REV: TTTACGTTGCGGCTTGTTAGG

AHR FWD: GGTTGTGATGCCAAAGGA; AHR REV: GGGACTCGGCACAATAAAG

CYP1A1 FWD: GGAGCTAGACACAGTGATTG; CYP1A1 REV: AAGAGTGTCGGAAGGTCT

TIPARP FWD: CTCGTGTTTGAGCTGGTGAA; TIPARP REV: ACACGTTCATGGCATTCAAA

IFNB1 FWD: CTTCTCCACTACAGCTCTTTC; IFNB1 REV: CTGTCCTTGAGGCAGTATTC

CXCL10 FWD: CTCCCATCACTTCCCTACA; CXCL10 REV: GGAGTAGTAGCAGCTGATTTG

CCL5 FWD: ACACCCTGCTGCTTTGCCTACA; CCL5 REV: TCCCGAACCCATTCTTCTCTG

ISG15 FWD: CATCTTTGCCAGTACAGGAG; ISG15 REV: ACACCTGGAATTCGTTGC

IRF1 FWD: GACTCCAGCTACAACAGATG; IRF1 REV: CTTCCCATCCACGTTTGT

#### RNA-seq

Total cellular RNA was isolated using the PureLink RNA Mini kit with on-column DNase treatment (Thermo Fisher Sceintific) and RNAseq analysis was carried on as previously described^73^. Briefly, sequencing libraries were prepared with the TruSeq Stranded mRNA kit (Illumina) from 500 ng total RNA following manufacturer’s instructions and single-end sequenced on a NextSeq 500 (Illumina) following the manufacturer’s recommended protocol. Genome alignment of raw reads to the human reference genome (GRCh38.p13) using STAR^74^ and raw feature count normalization were performed at the Roswell Park Comprehensive Cancer Center’s Genomics Shared Resource. Differential expression analysis was carried out with DESeq2^75^ and differential expression rank order was used for subsequent gene set enrichment analysis (GSEA)^76^, performed using the cluster profile package in R and collections available through the Molecular Signatures Database (MSigDB)^77^. Overlaps of DEG lists across companions were calculated by hypergeometric testing. All analyses were performed using R statistical software, version 4.1.1.

### Immunoblotting

Whole cell lysates were collected using RIPA buffer (50 mM Tris–HCl, 150 mM NaCl, 1% Triton X-100, 0.5% sodium deoxycholate, 0.1% SDS, 10% glycerol, 2 mM EDTA) and separated by SDS-PAGE on polyacrylamide gel (8–15% acrylamide depending on protein targets). Proteins were transferred to nitrocellulose membranes (Biorad, Hercules, CA, USA) and membranes were blocked in Blocking buffer (5% milk in TBS containing 0.1% Tween-20) for 1 h at room temperature. Membranes were incubated with primary antibody diluted in blocking buffer overnight at 4 °C. Appropriate HRP-conjugated secondary antibodies (Bio-Rad, Hercules, CA, USA) were used at 1:5,000 dilution in blocking buffer for 1 h at RT. Signals were visualized with BioRad chemiluminescence reagents and either xray films developed with a Konica SRX-101A (Konica Minolta, Wayne, NJ, USA) film processor or with a GeneGnome XRQ NPC system (Syngene, Frederick, MD, USA). Densitometric quantifications were performed with ImageJ (National Institutes of Health, Bethesda, MD, USA). Membranes were cut prior to hybridization with antibodies.

### Supplementary Information

Supplementary Information 1.

Supplementary Information 2.

Supplementary Information 3.

## Data Availability

The data that support the findings of this study are available from the corresponding authors upon reasonable request. All sequencing data reported here are available in the NCBI Gene Expression Omnibus (GEO) database under GSE252119.
