# Supplementary material for: Aryl hydrocarbon receptor suppresses STING-mediated type I IFN expression in triple-negative breast cancer
Source: Sci Rep. 2024 Mar 8;14:5731. doi: 10.1038/s41598-024-54732-3 (PMC10923803; doi:10.1038/s41598-024-54732-3)
Supplement: Supplementary file 1 — Supplementary Information 1. [file 41598_2024_54732_MOESM1_ESM.docx]

**SUPPLEMENTAL FIGURE LEGENDS**

**Supplemental Figure 1. AHR is not a negative prognostic indicator in Luminal A/B or HER2-amp breast cancer** **A-C.** Kaplan Meier analysis for overall survival of breast cancer patients (panel A. Luminal A, panel B. Luminal B, panel C. HER2-positive), stratified on median level of AHR expression performed at https://kmplot.com. Statistics by LogRank Test.

**Supplemental Figure 2. RNA sequencing in AHR-knockdown MDA-MB-231 cells reveals interferon-related signatures.** Gene set enrichment analysis for differentially expressed genes in MDA-MB-231 control and AHR-depleted.

**Supplemental Figure 3. AHR suppresses IFN-I expression in TNBC cells but not MCF10A or WI-38 cells. A.** qRT-PCR analysis of indicated genes in HCC1937 cells (20µM BAY2416469, 48 hours). Data (n=3 biological replicates) is average -/+ std dev. Statistics by 2-way ANOVA. **B&C.** qRT-PCR analysis of indicated genes in MCF10A (panel B) and WI-38 (panel C) cells (20µM BAY2416469, 48 hours). Data (n=3 biological replicates) is average -/+ std dev. Statistics by 2-way ANOVA. * p<0.05. ** p<0.01, *** p<0.005, ****p<0.0005.

**Supplemental Figure 4. MDA-MB-231 and MDA-MB-436 cells display robust AHR activity in response to the AHR agonist TCDD at short time point.** MDA-MB-231 (**A**) or MDA-MB-436 (**B**) cells were treated with 50nM TCDD for 2 hours. RNA was collected and qPCR was performed for CYP1A1 and TIPARP (n = 3 technical replicates).

**Supplemental Figure 5. AHR antagonist-mediated IFN-I expression is STING-dependent. A**. qRT-PCR analysis of indicated genes in MDA-MB-436 cells (pLKO = white bars, shSTING = grey bars – 20µM BAY, 72 hours). Data (n=3 biological replicates) is average -/+ std dev. Statistics by 2-way ANOVA. **B.** qRT-PCR analysis of indicated genes in MDA-MB-436 cells (10µM H-151, 20µM BAY2416964, or the combination, 72 hours). Data (n=2 biological replicates) is average -/+ std dev. Statistics by 2-way ANOVA **C.** MDA-MB-436 cells were treated with 10µM H-151, 20µM BAY2416964, or the combination, 72 hours. Quantification of relative p-TBK1 (S172) densitometry, normalized to ß-actin. Data (n=3 biological replicates) is average -/+ std dev. Statistics by 2-way ANOVA. * p<0.05. ** p<0.01, *** p<0.005.

**Supplemental Figure 6. BRCA1 knockdown in MDA-MB-231 cells. A**. Cell viability analysis in MDA-MB-231 cells (pLKO – black line, shBRCA1 – red line) treated with increasing concentrations of Talazoparib for 96 hours. Data (n=3 biological replicates) is average -/+ std dev.
