# Supplementary figures and images for: Aryl hydrocarbon receptor suppresses STING-mediated type I IFN expression in triple-negative breast cancer
Source: Sci Rep. 2024 Mar 8;14:5731. doi: 10.1038/s41598-024-54732-3 (PMC10923803; doi:10.1038/s41598-024-54732-3)

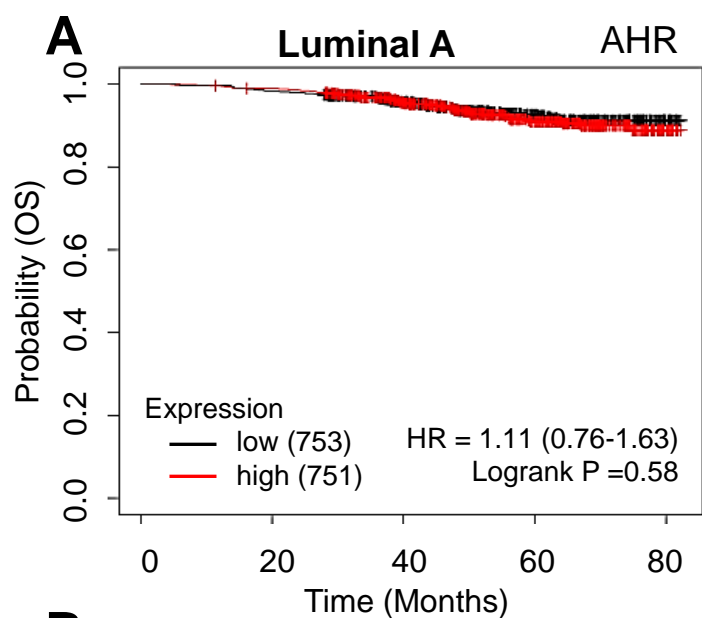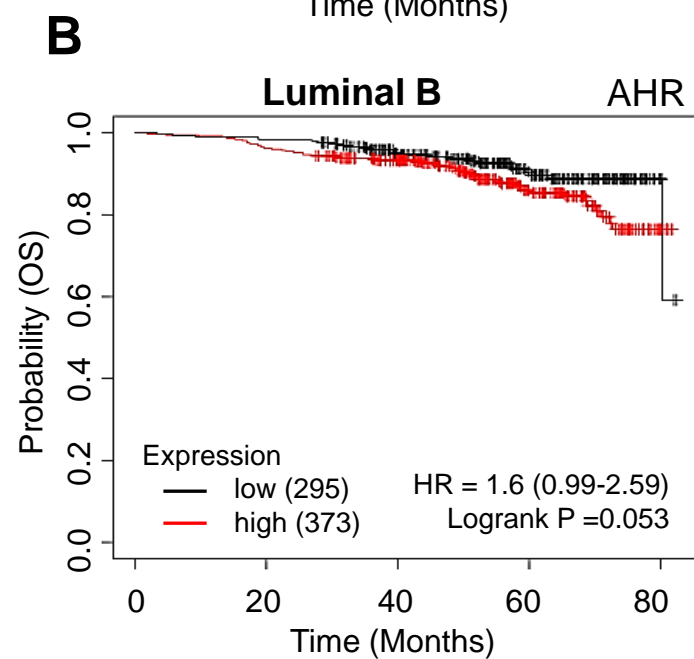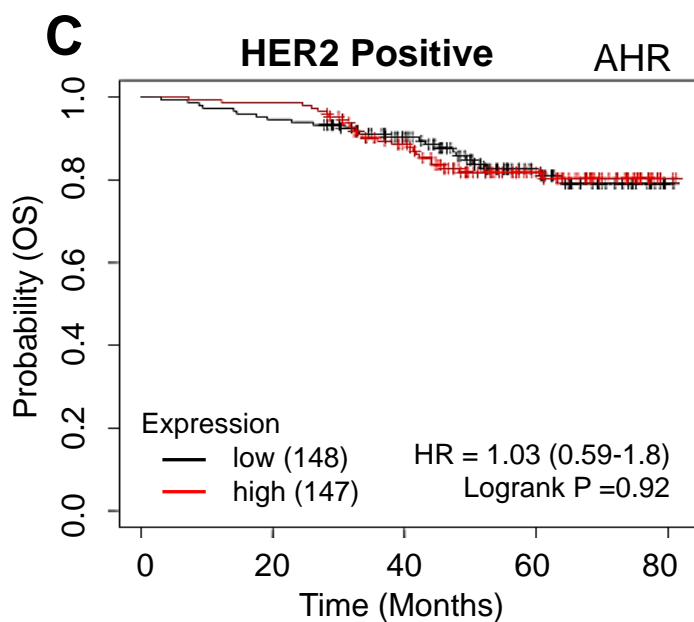

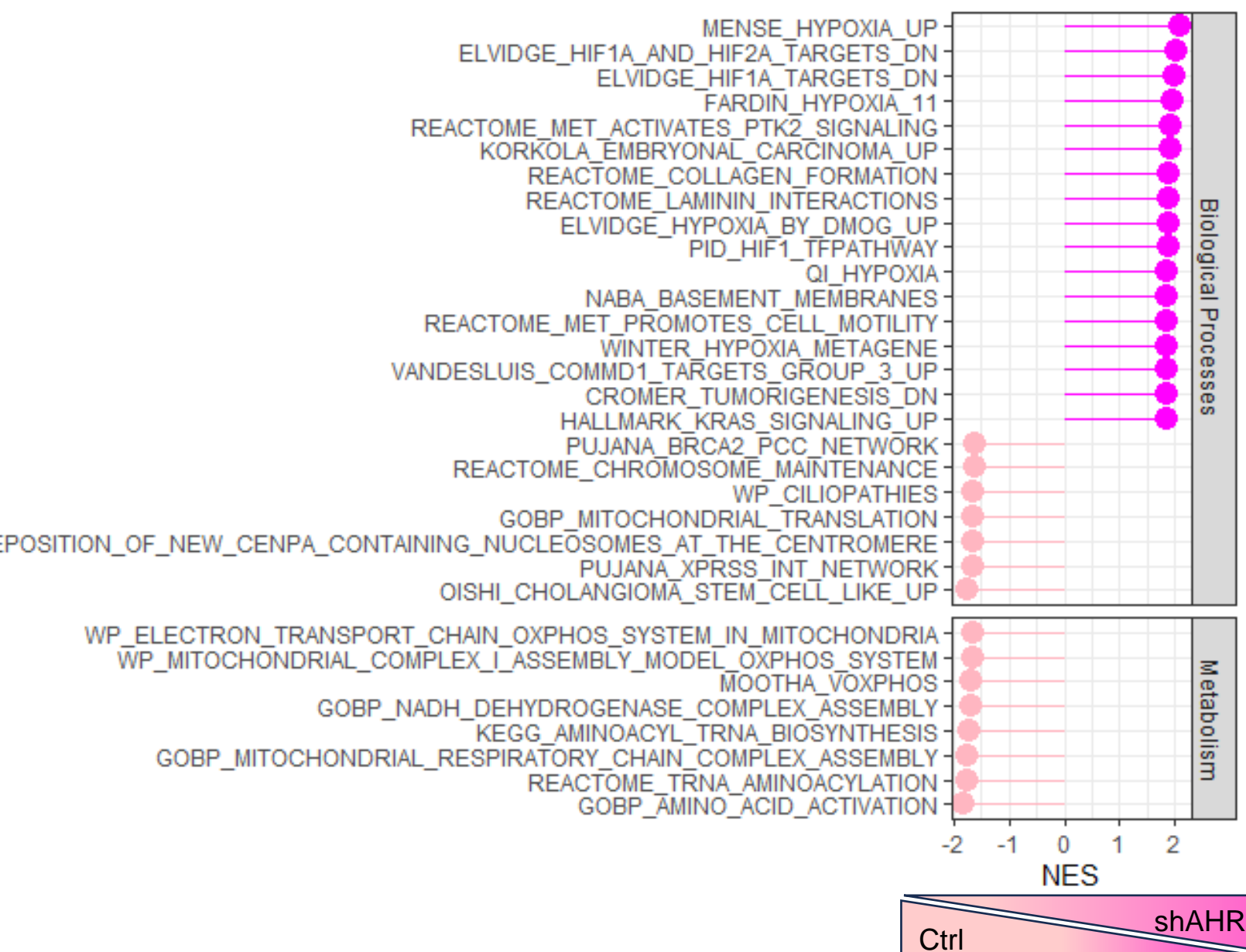

Supplemental Figure S2

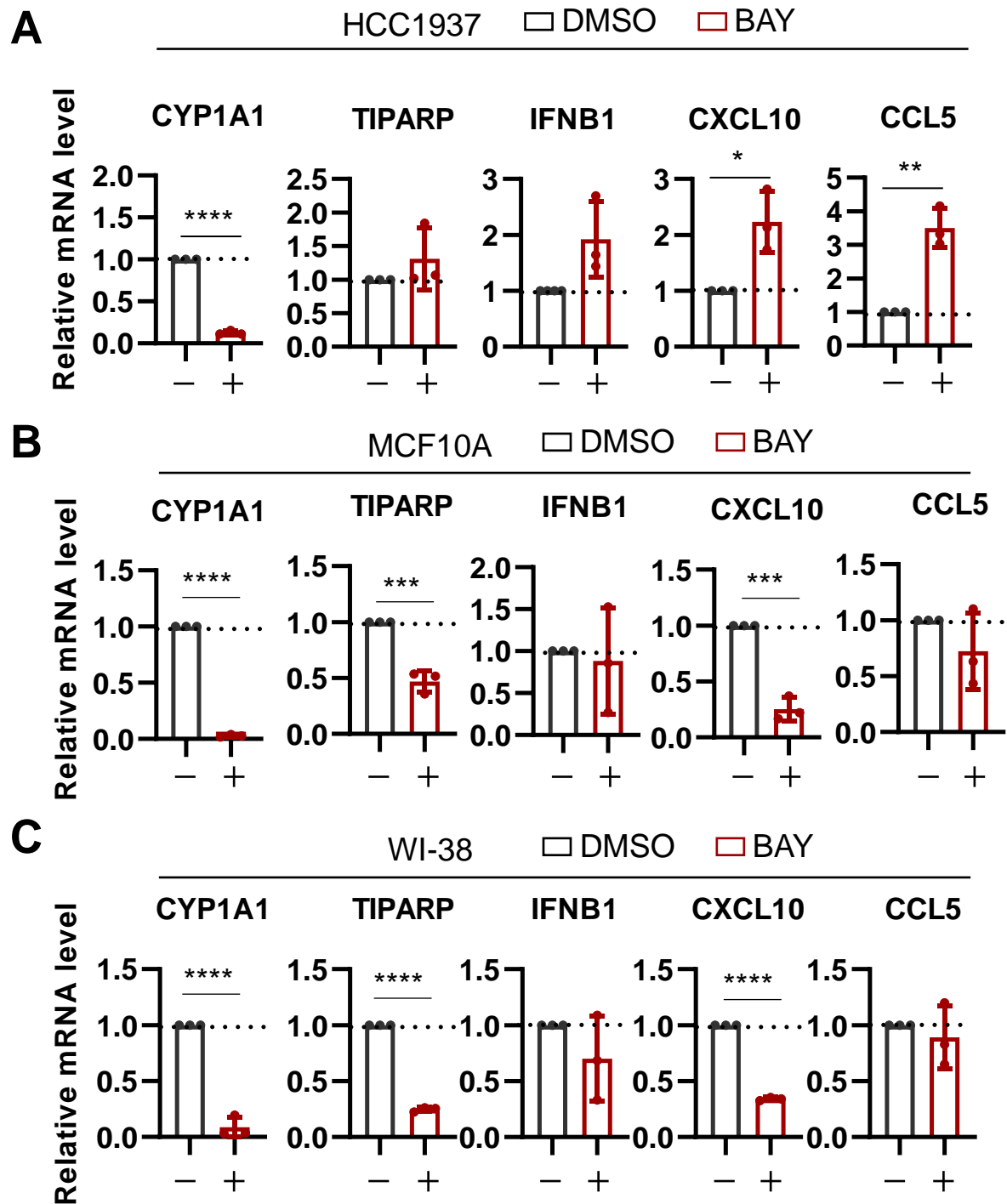

**A**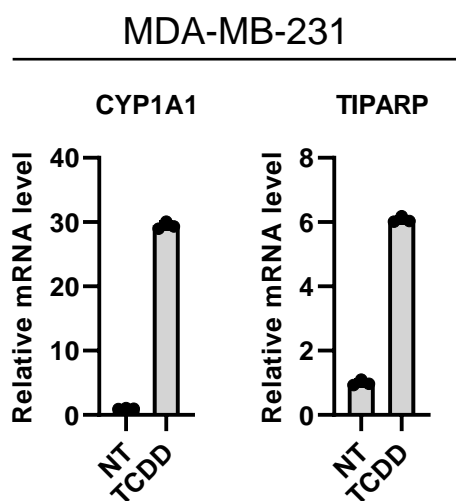**B**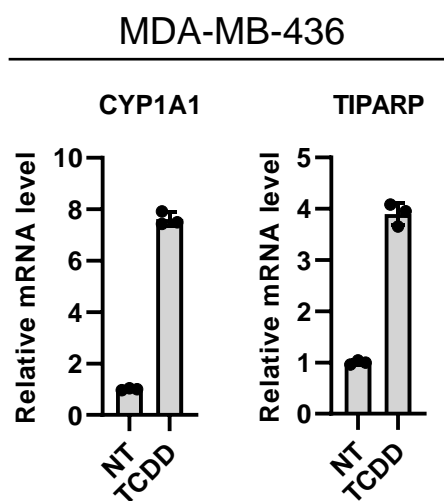

**A**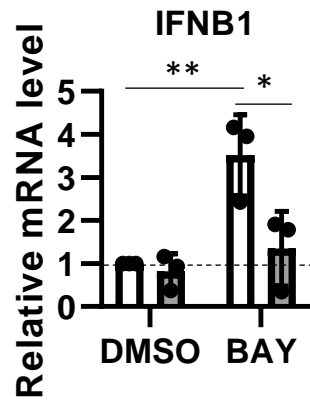**B**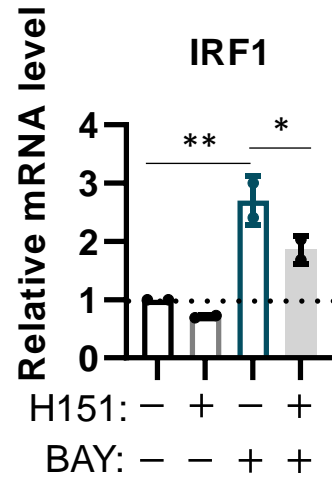**C**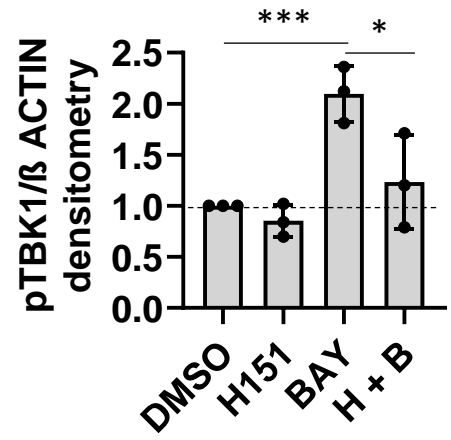

**A**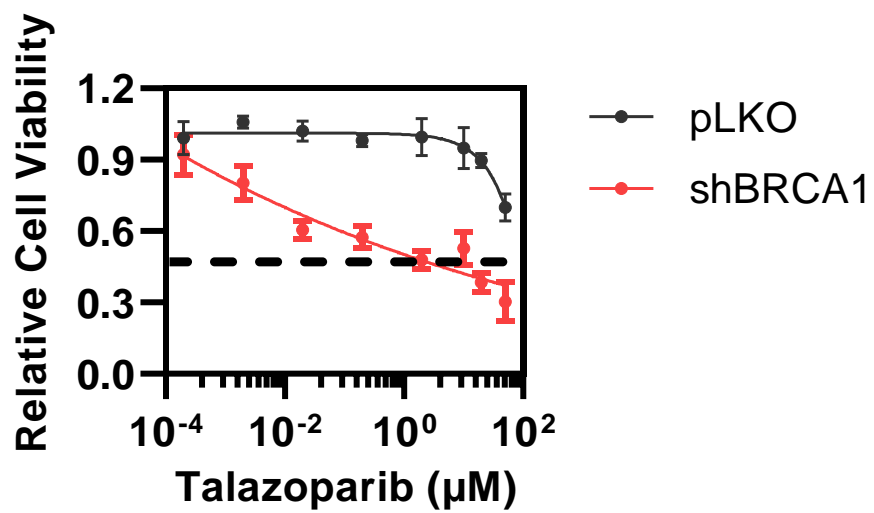

Supplement: Supplementary file 2 — Supplementary Information 2. [file 41598_2024_54732_MOESM2_ESM.pdf]
