# Supplementary material for: Aryl hydrocarbon receptor suppresses STING-mediated type I IFN expression in triple-negative breast cancer
Source: Sci Rep. 2024 Mar 8;14:5731. doi: 10.1038/s41598-024-54732-3 (PMC10923803; doi:10.1038/s41598-024-54732-3)

Membrane edges identified w/ red arrows

AHR figure 2C

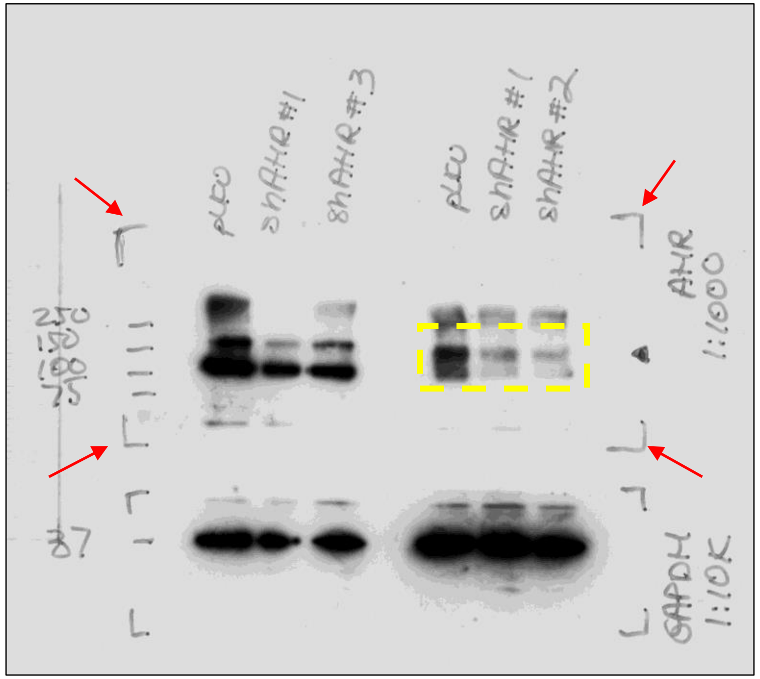

GAPDH figure 2C

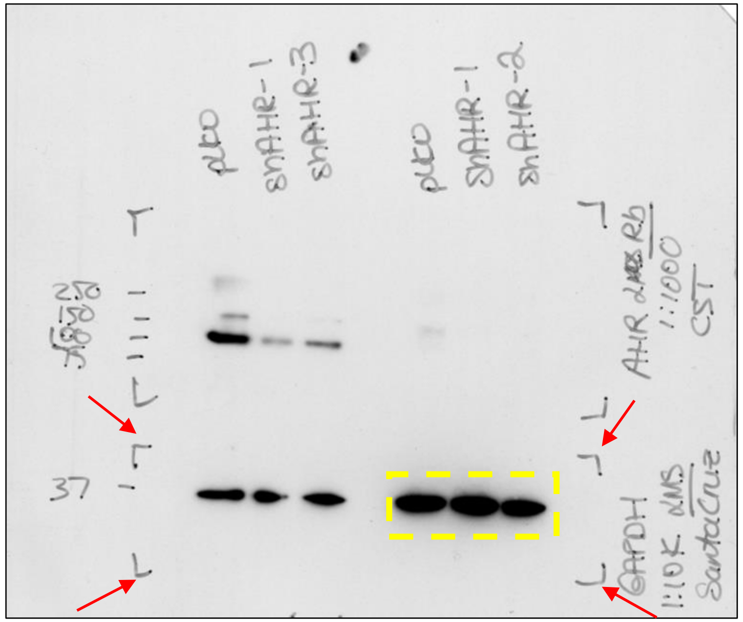

MDA-MB-436

Membrane edges identified w/ red lines

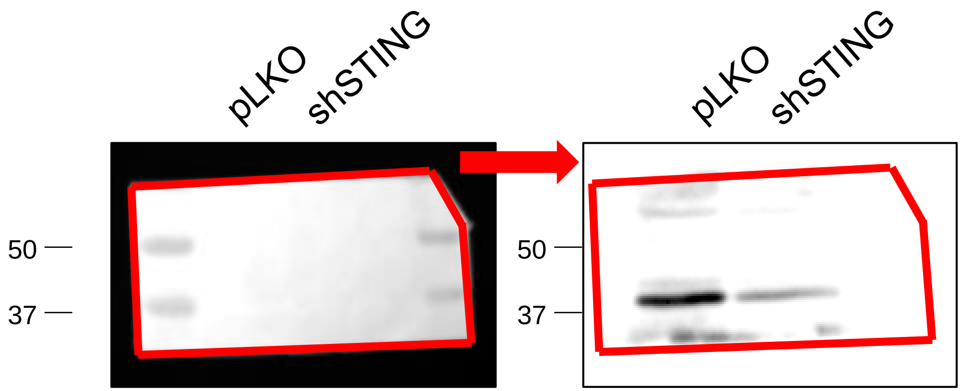

STING for figure 3A

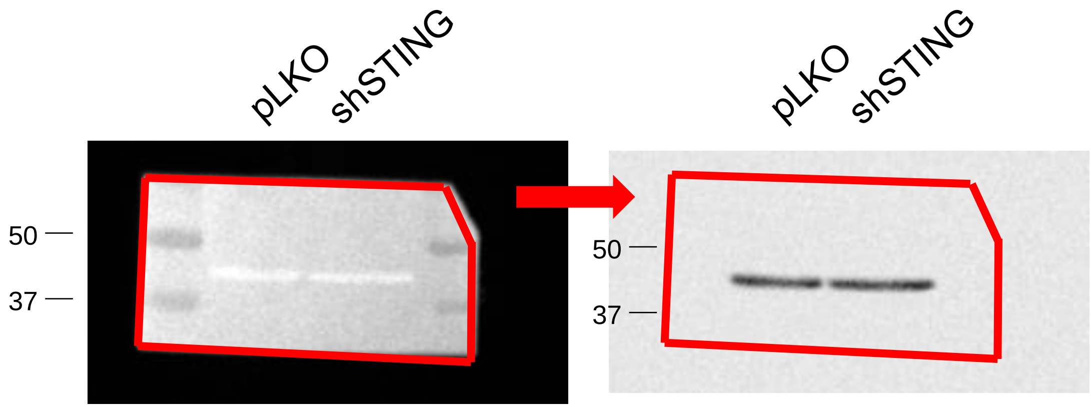

ß-Actin for figure 3A

Membrane edges identified w/ red arrows

pTBK1
MW = ~85kDa

pTBK1 (S172) figure 3D

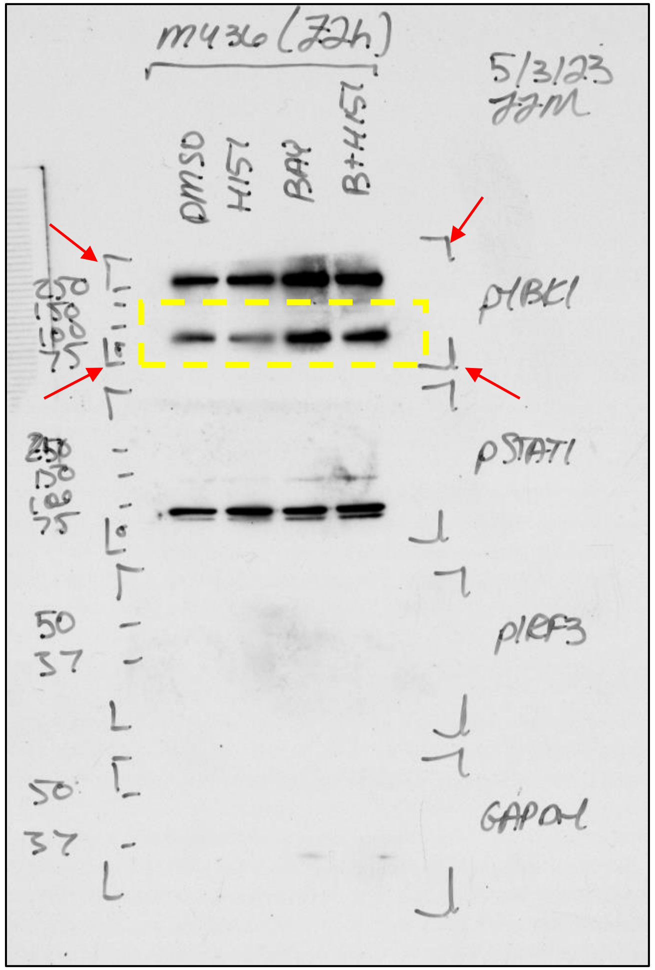

ß ACTIN figure 3D

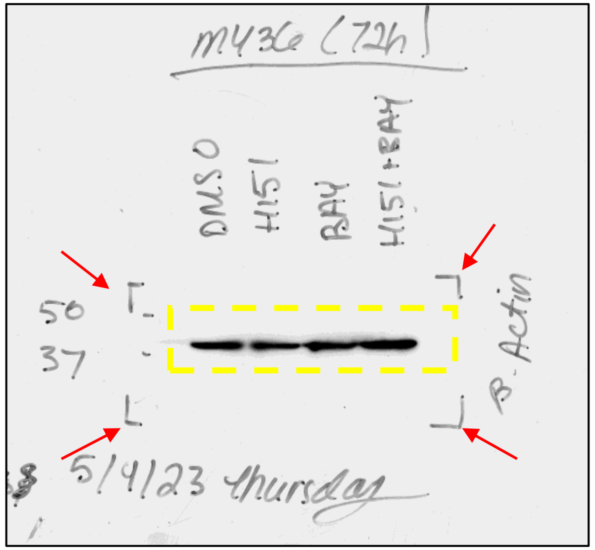

Total TBK1 figure 3D

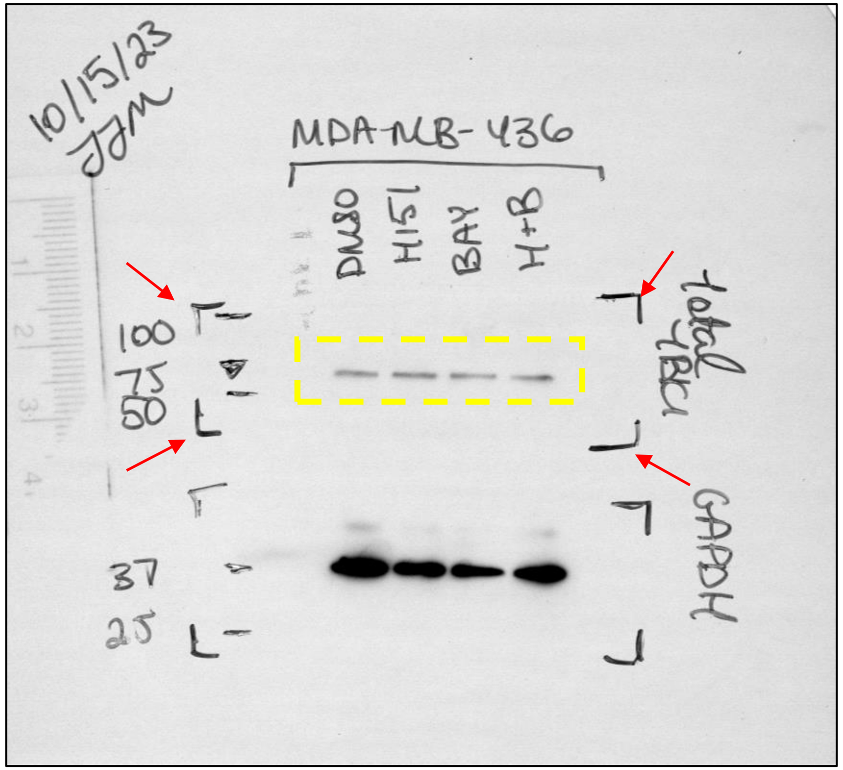

Membrane edges identified w/ red arrows

BRCA1 for Figure 4B

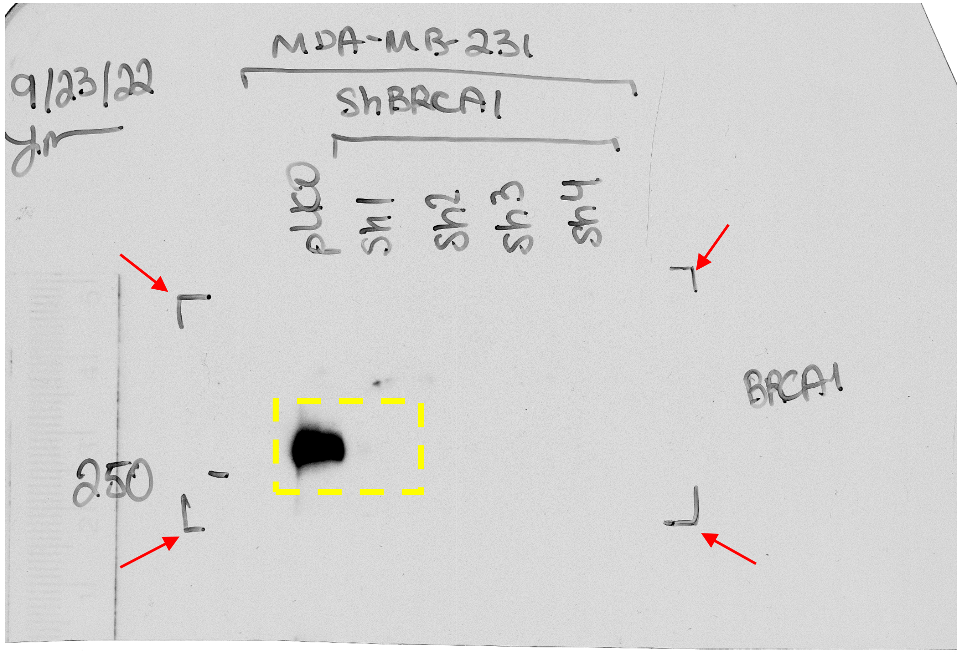

Vinculin for Figure 4B

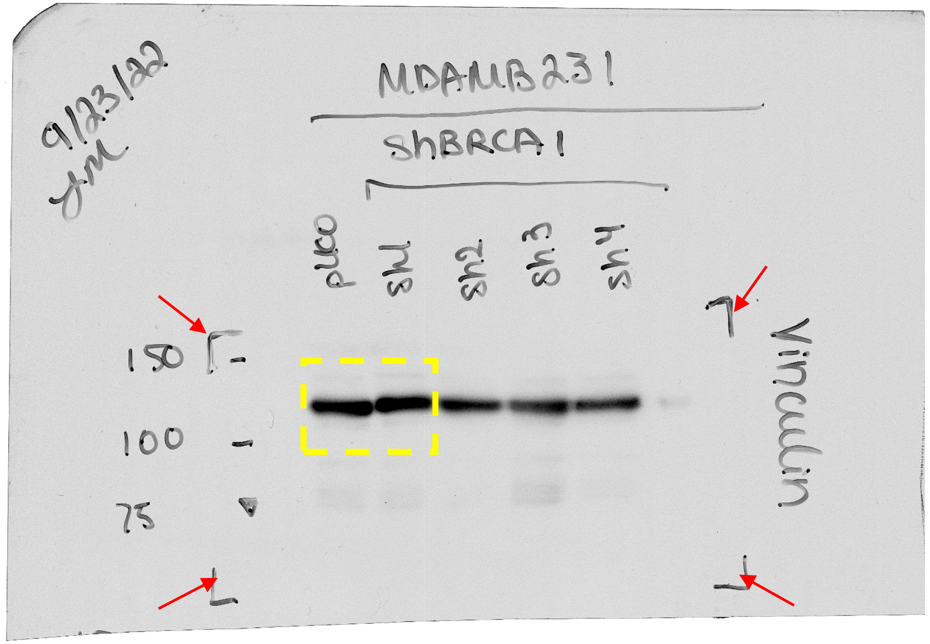

Membrane edges identified w/ red arrows

Supplemental Figure S5C
M436 cells were treated with 20µM BAY or 10µM H-151 for 72 hours – TRIAL 1
*****SHOWN IN FIGURE 3D*****

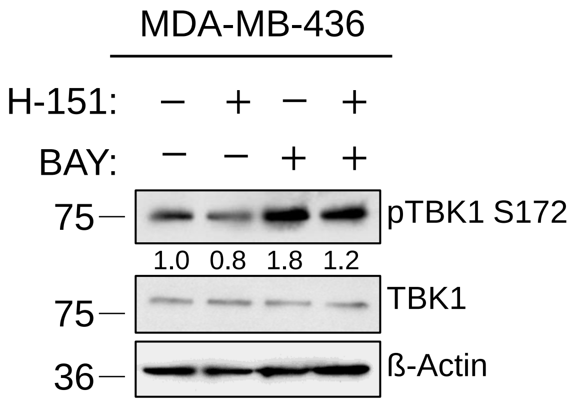

pTBK1 (S172) figure 3C

pTBK1
MW = ~85kDa

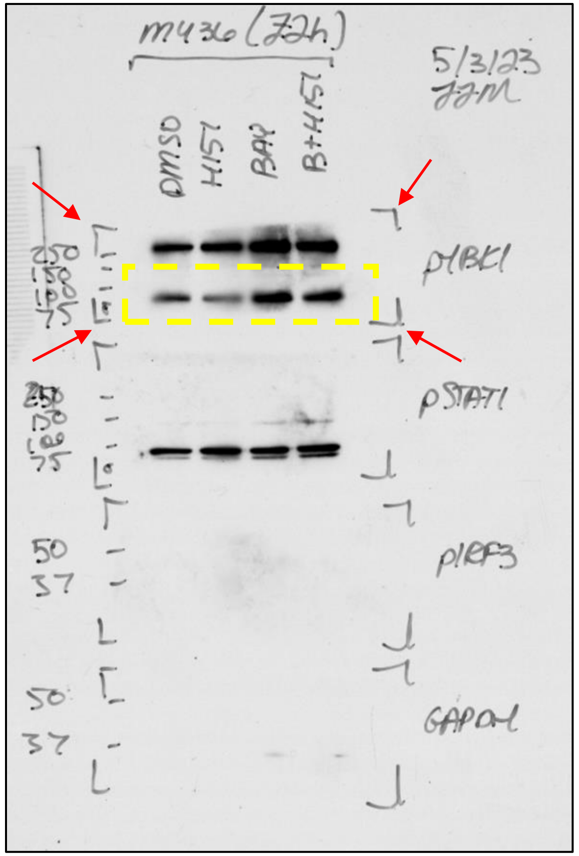

ß ACTIN figure 3C

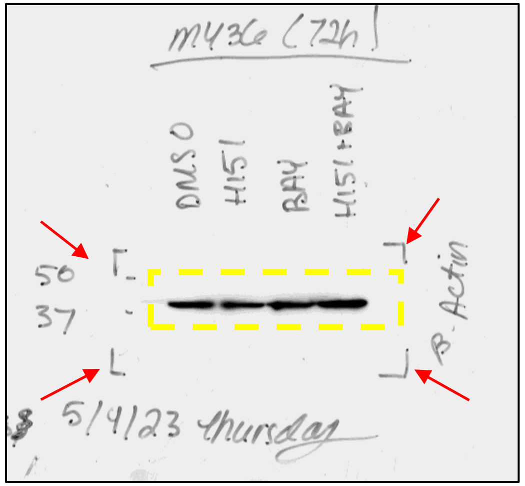

Total TBK1 figure 3C

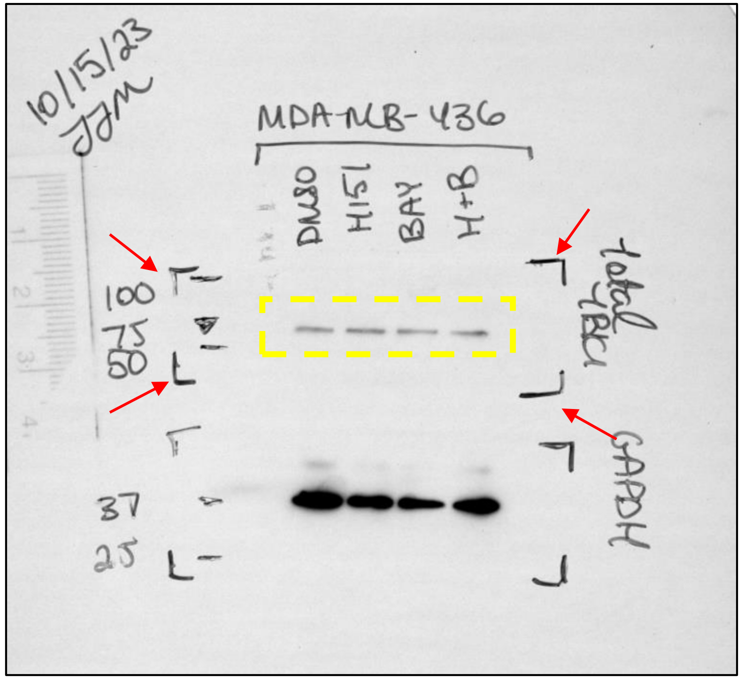

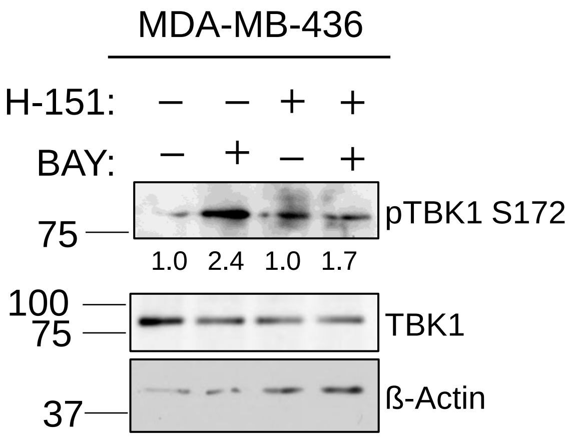

Supplemental Figure S5C M436 cells were treated with 20µM BAY or 10µM H-151 for 72 hours – TRIAL 2

Membrane edges identified w/ red arrows/lines

pTBK1 S172

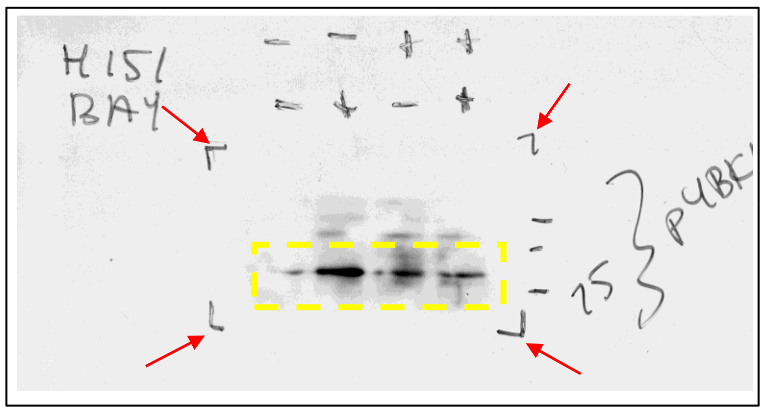

ß-Actin

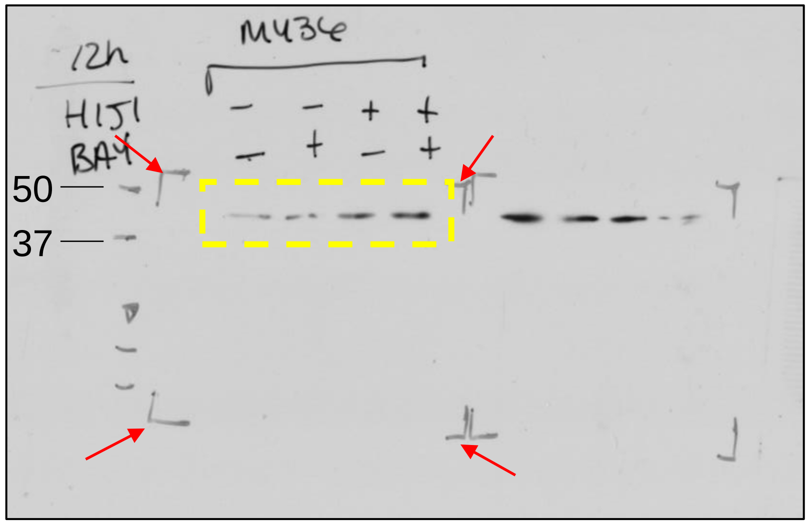

TBK1

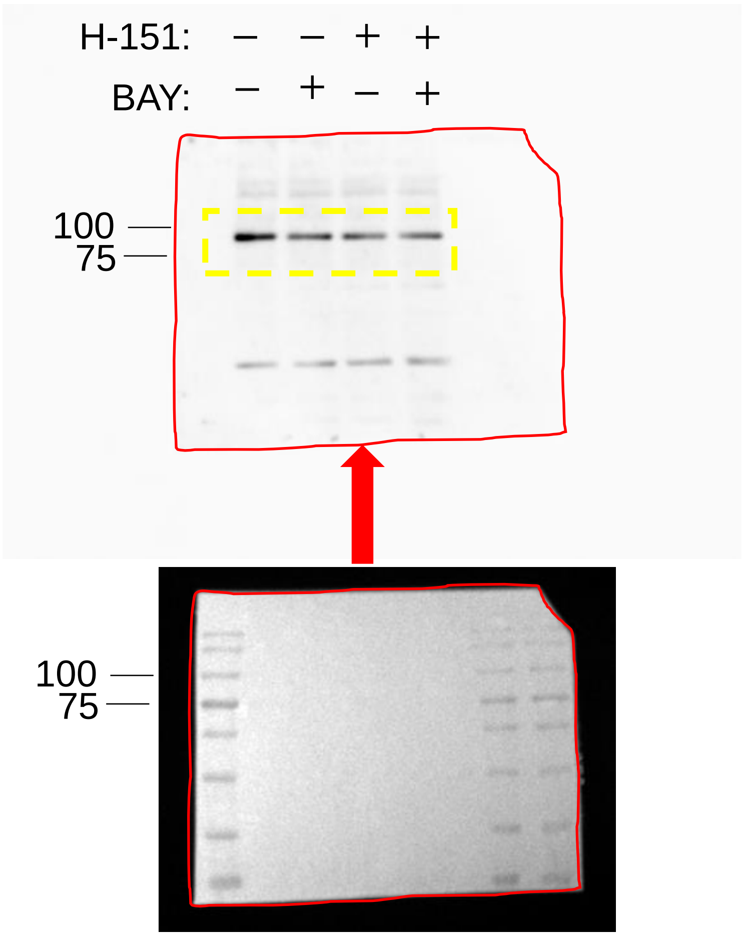

Supplemental Figure S5C M436 cells were treated with 20µM BAY or 10µM H-151 for 72 hours – TRIAL 3

Membrane edges identified w/ red lines

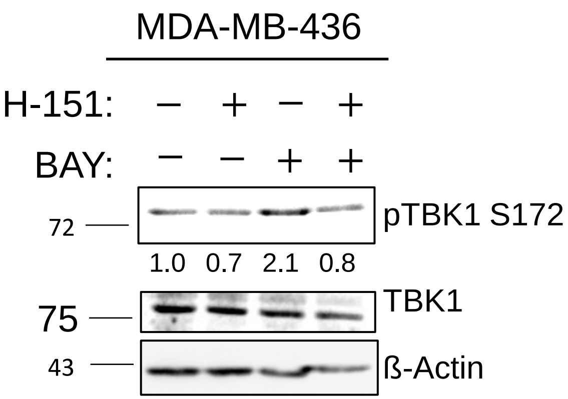

ß-Actin

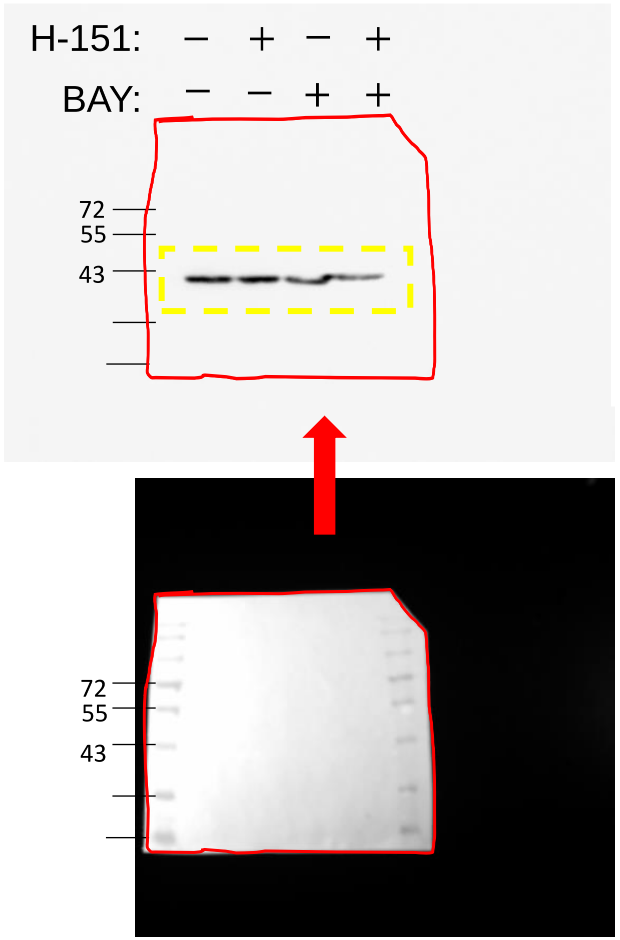

pTBK1 S172

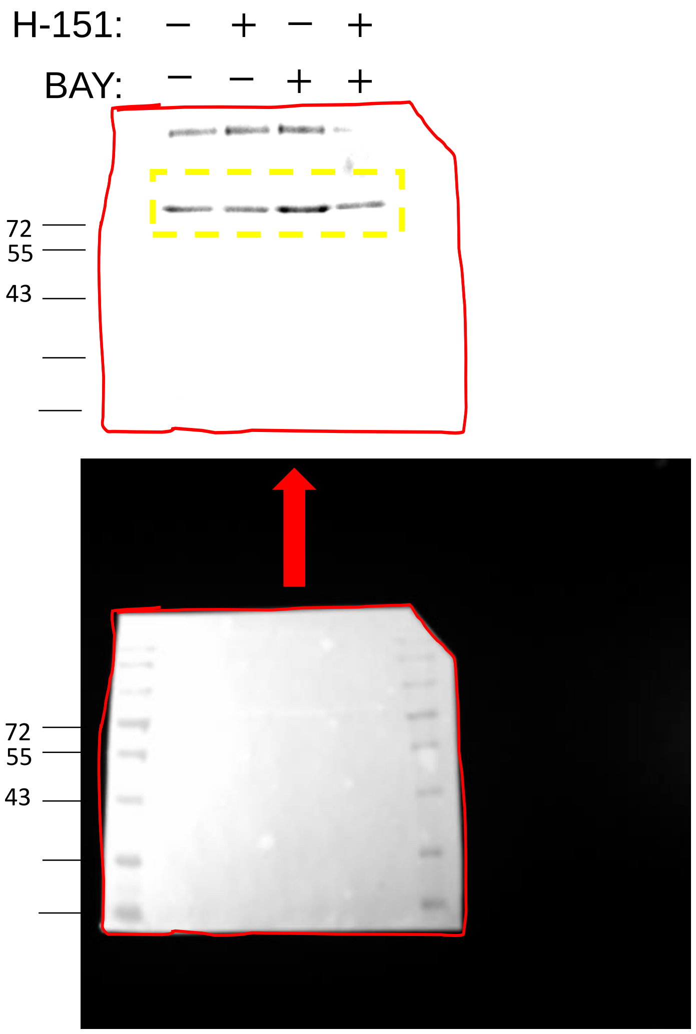

TBK1

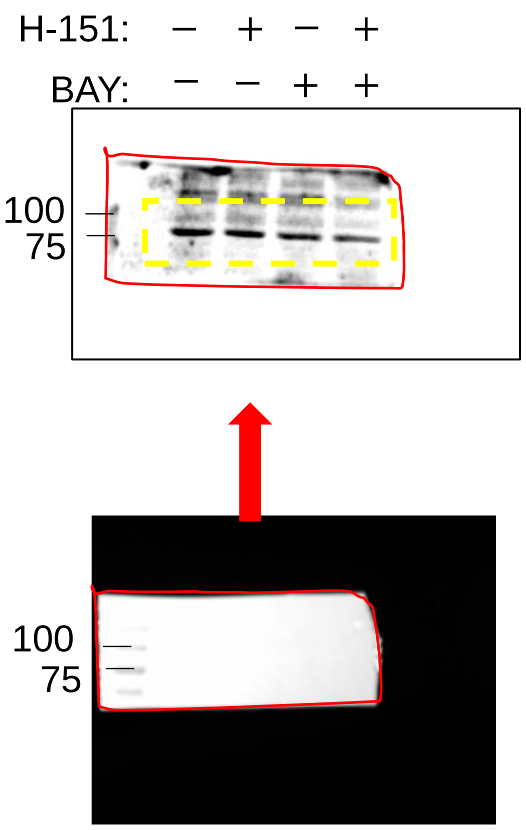

Supplement: Supplementary file 3 — Supplementary Information 3. [file 41598_2024_54732_MOESM3_ESM.pdf]
